# Prevalence and trend of smokeless tobacco use and its associated factors among adolescents aged 12–16 years in 138 countries/territories, 1999–2019

**DOI:** 10.1186/s12916-022-02662-0

**Published:** 2022-11-25

**Authors:** Hui Yang, Chuanwei Ma, Min Zhao, Costan G. Magnussen, Bo Xi

**Affiliations:** 1 Department of Epidemiology, School of Public Health, Qilu Hospital, Cheeloo College of Medicine, Shandong University, Jinan, China; 2 Department of Nutrition and Food Hygiene, School of Public Health, Cheeloo College of Medicine, Shandong University, Jinan, China; 3 Baker Heart and Diabetes Institute, Melbourne, VIC Australia; 4 Research Centre of Applied and Preventive Cardiovascular Medicine, University of Turku, Turku, Finland; 5 Centre for Population Health Research, University of Turku and Turku University Hospital, Turku, Finland

**Keywords:** Smokeless tobacco use, Trends, Factors, Adolescents

## Abstract

**Background:**

Smokeless tobacco use is popular in some regions worldwide, but it receives less attention compared to cigarette smoking. We aimed to estimate the recent prevalence of, and trends in, smokeless tobacco use and to examine its associated factors among adolescents aged 12–16 years in 138 countries/territories (hereafter “countries”) from 1999 to 2019.

**Methods:**

Data from the Global Youth Tobacco Survey conducted in 138 countries in 2010–2019 and the National Youth Tobacco Survey conducted in the United States in 2019 were used to calculate the prevalence of current smokeless tobacco use and investigate its associated factors among adolescents aged 12–16 years. We also assessed the trend in the prevalence of smokeless tobacco use in 100 countries that had conducted more than one survey from 1999 to 2019.

**Results:**

The overall prevalence of current smokeless tobacco use was 4.4% (95% confidence interval [CI] 4.0–4.9), with 5.7% (5.1–6.3) for boys, 3.1% (2.6–3.5) for girls, 3.9% (3.5–4.4) for adolescents aged 12–14 years and 5.4% (4.8–5.9) for those aged 15–16 years. The prevalence was highest in the South-East Asian region (6.1%, 4.4–7.7) and lowest in the Western Pacific region (2.0%, 1.7–2.4). The prevalence of smokeless tobacco use decreased in 57 of 100 countries, increased in 32 countries, and remained unchanged in 11 countries. Current cigarette smoking (odds ratio [OR]=2.00, 95% CI=1.68–2.39), other tobacco product use (OR=6.03, 95% CI=4.92–7.40), tobacco advertisement exposure (OR=1.44, 95% CI=1.19–1.74), being offered free tobacco products (OR=2.01, 95% CI=1.66–2.42), and not being taught about dangers of smoking (OR=1.28, 95% CI=1.09–1.50) were all positively associated with current smokeless tobacco use.

**Conclusions:**

Smokeless tobacco use among adolescents remains a public health concern worldwide. Although the prevalence among adolescents decreased in most countries, it remains high especially in the South-East Asian region. More strict and effective strategies and measures are needed to further curb the smokeless tobacco use among adolescents.

**Supplementary Information:**

The online version contains supplementary material available at 10.1186/s12916-022-02662-0.

## Background

Smokeless tobacco is a type of tobacco that is consumed by chewing, sucking, snuffing, or other non-combustion forms. Historically, smokeless tobacco was popular mainly in the South-East Asian region. However, due to the implementation of smoke-free policies [1] and the diversification of tobacco market worldwide [2–5], the increasing use of smokeless tobacco products has become a growing global health concern. For example, there were about 356 million smokeless tobacco users worldwide by 2015, with nearly 82% living in South-East Asia [6]. Globally, smokeless tobacco use was responsible for over 650,000 deaths in 2010 (accounting for approximately 10% of all deaths due to all forms of tobacco use) [7], and an estimated 315,000 deaths in 2016 [8]. In 2017, the global burden of disease caused by smokeless tobacco use amounted to approximately 8.7 million disability adjusted life years [9]. Although smokeless tobacco is thought to be less harmful to health than burnt cigarettes, and considered a smoke-free alternative, smokeless tobacco products contain more than 30 harmful compounds that have been linked to nicotine addiction [10, 11], nicotine poisoning [12], oral disease [10, 13], adverse pregnancy outcomes [11, 14], diabetes [15], cardiovascular disease [10, 13], and cancers [10], which contribute substantially to the global disease burden [9].

The majority of tobacco users usually start smoking during adolescence, and adolescents are more vulnerable to the harmful effects of tobacco products use because they are prone to addiction to the nicotine in tobacco [16]. There is an apparent increase in the use of smokeless tobacco products among adolescents [17, 18], but its recent epidemic pattern is poorly understood. Based on data from the Global Youth Tobacco Survey (GYTS) in 75 countries in 2007–2010, the prevalence of smokeless tobacco use among adolescents aged 13–15 years ranged from 1.1% in Montenegro to 16.4% in Congo, and the prevalence was >10% in 12 countries for boys and in four countries for girls [19]. According to the report from the World Health Organization (WHO) Framework Convention on Tobacco Control (FCTC), the overall prevalence of smokeless tobacco use among adolescents aged 13–15 years was 6.6% for boys and 3.6% for girls based on data from various sources in 106 countries prior to 2015 [18]. Using the GYTS data conducted in 131 countries in 2007–2016, Cahn et al. estimated that nearly 13 million adolescents aged 13–15 years were current smokeless tobacco users, and the prevalence was highest (7.45%) in the African region, and lowest (2.0%) in the European region [8]. Difference in the prevalence of smokeless tobacco use across countries or regions might be due to differences in social acceptance and implementation of FCTC provisions on smokeless tobacco [19–21]. In addition, several previous studies from different countries showed that initiation of smokeless tobacco use among adolescents was associated with a variety of influencing factors, such as cigarette smoking and social environmental factors [22–24]. On the other hand, exclusive smokeless tobacco use among youth also contributes to the initiation of cigarette smoking and poly-tobacco use during adolescence and adulthood [25, 26], and increases the risk of tobacco-related chronic disease and mortality in adults [27]. To our knowledge, no previous studies have assessed the associated factors of, and trend in, smokeless tobacco use among adolescents aged 12–16 years based on global data.

In this study, we aimed to estimate the recent global prevalence of smokeless tobacco use among adolescents aged 12–16 years and to examine its associated factors based on the latest GYTS data conducted in 2010–2019 in 138 countries/territories (hereafter “countries”) and the National Youth Tobacco Survey (NYTS) data conducted in the United States (U.S.) in 2019. We also determined the secular trend in the prevalence of smokeless tobacco use among adolescents in 100 countries from 1999 to 2019.

## Methods

### Study design and participants

Data on smokeless tobacco use were extracted from the GYTS, which is a cross-sectional and school-based program led by the WHO and the U.S. Centers for Disease Control and Prevention aiming to provide nationally representative estimates on tobacco use among adolescents and guide tobacco control programs worldwide. The GYTS used the same two-stage random cluster sampling framework in each participating country, and at the first stage, schools were randomly selected, and at the second stage, classes were also randomly selected from the target schools. All students in the selected classes were eligible to complete the standardized questionnaires, which were self-administered by students and included the same core set of questions to make them comparable across countries. More information on the GYTS can be found on the Centers for Disease Control and Prevention website (https://www.cdc.gov/tobacco/global/gtss/gtssdata/index.html) [28].

Data in the U.S. were from the NYTS, which is a national, annual, and school-based program to assess tobacco use among U.S. youth. The NYTS followed a similar methodology to the GYTS. The questions and possible closed-ended answers on smokeless tobacco use and related factors are consistent with the GYTS. More information on the NYTS can be found on the U.S. Centers for Disease Control and Prevention website (https://www.cdc.gov/tobacco/data_statistics/surveys/nyts/index.htm) [29]. All GYTSs and NYTSs were approved by the National Ethics Committee, and all students/guardians provided verbal consent.

Both the GYTS and NYTS are ongoing. We used the latest data from 138 countries in 2010-2019 to estimate the prevalence of smokeless tobacco use and its associated factors among adolescents aged 12-16 years. We also used data from 100 countries that had conducted two or more surveys between 1999 and 2019 to estimate the secular trend in the prevalence of smokeless tobacco use. Of note, the GYTS did not release the latest data in China, thus we extracted the related information from the Chinese Youth Tobacco Survey report in 2014 [30], which is a part of the GYTS. A flow chart of inclusion and exclusion of countries is shown in Additional file 1: Fig. S1. After excluding participants with missing data on sex, age and current smokeless tobacco use, and those aged <12 or >16 years, a total of 1,039,249 adolescents aged 12–16 years between 1999 and 2019 were included in the data analyses.

### Dependent variable

Current smokeless tobacco use was defined as using any form of smokeless tobacco products during the past 30 days based on response to the question “During the past 30 days, did you use any form of smokeless tobacco products (such as snuff, chewing tobacco, dip, gutka)?”.

### Independent variable

Demographic variables included sex and age. Other independent variables included cigarette smoking, other tobacco product use, parental smoking, smoking status of closest friends, tobacco advertisement exposure, being offered free tobacco products, being taught about dangers of smoking, and World Bank income level. Cigarette smoking was defined as smoking cigarettes on at least one day during the past 30 days based on response to the question “During the past 30 days, on how many days did you smoke cigarettes?”. Other tobacco product use was defined as using combustible tobacco products other than cigarettes during the past 30 days based on response to the question “During the past 30 days, did you use any form of smoked tobacco products other than cigarettes (such as cigars, pipe, waterpipe)?”. Parental smoking was assessed based on response to the question “Do your parents smoke tobacco?”, and it was divided into “Neither smoking,” “Only father smoking,” “Only mother smoking,” and “Both smoking” in our analyses. Smoking status of closest friends was assessed based on response to the question “Do any of your closest friend smoke tobacco?”, and it was divided into “None”, “Some”, “Most”, and “All”. Tobacco advertisement exposure was defined as exposure to more than one type of tobacco advertisement based on responses to the following three questions: “During the past 30 days, did you see any people using tobacco on TV, in videos, or in movies?”, “During the past 30 days, did you see any advertisements or promotions for tobacco products at points of sale (such as stores, shops, restaurant)?”, and “Do you have something with a tobacco product brand logo on it (such as t-shirt, pen, backpack)?”. Being offered free tobacco products was assessed based on response to the question: “Has a person working for a tobacco company ever offered you a free tobacco product?”. Being taught about dangers of smoking was assessed based on response to the question: “During the past 12 months, were you taught in any of your classes about the dangers of tobacco use?”. Income level categories for each country followed the standards of the World Bank's classification based on the survey year of the GYTS/NYTS in our study.

### Statistical analysis

According to the complex sampling design of the GYTS, the weighted prevalence estimates and their 95% confidence intervals (CI) of smokeless tobacco use in each country were calculated using original sampling weights, strata, and primary sampling units provided in the datasets using the SAS PROC SURVEYFREQ procedure. The original weights were calculated by the following formula: W=W_1_*W_2_*f_1_*f_2_*f_3_*f_4_, where W_1_ is the inverse of the selection probability of each school; W_2_ is the inverse of selection probability of each class; f_1_ is the school-level non-response adjustment factor calculated by school enrolment size; f_2_ is the class-level non-response adjustment factor for each school; f_3_ is the student-level non-response adjustment factor for each class; f_4_ is the post-adjustment stratification factor calculated by grade and sex. We rescaled the original weights to calculate the overall and subgroups’ prevalence of smokeless tobacco use based on each country’s sample size. The rescaled weights were calculated as the maximum country sample size (i.e., U.S.: *n*=13,689) divided by the sample size of each country multiplied by the original weights. Chi-square analysis was used to test for differences in the prevalence between sexes, age groups, and other subgroups. Chi-square trend test was used to examine the secular trend in the prevalence with consideration of data from all surveys in each country between 1999 and 2019. The prevalence estimates of secular trends were calculated per 5 calendar years. Multivariable logistic regression models were used to assess the association of potential associated factors (sex, age, cigarette smoking, other tobacco product use, parental smoking, smoking status of closest friends, tobacco advertisement exposure, being offered free tobacco products, being taught about dangers of smoking, and World Bank income level) with current smokeless tobacco use, and the code for each variable is shown in Additional file 1: Table S1. In order to correct the overall probability of type I error in multiple statistical tests (*α*=0.05), the Bonferroni’s correction was used to adjust the critical significance level of each statistical test. The corrected critical significance level was equal to the original critical significance level (0.05) divided by the number of tests performed. A two-sided *P*-value less than the Bonferroni’s corrected critical significance level was regarded as statistical significance and SAS 9.4 (SAS Institute, Cary, NC, US) was used for all analyses.

## Results

A total of 450,691 adolescents (boys: 51.4%) aged 12–16 years from 138 countries surveyed between 2010 and 2019 were included to estimate the prevalence of current smokeless tobacco use and its associated factors. Among 138 included countries surveyed in 2010–2019, 23 (16.7%) were from the African region, 30 (21.7%) from the American region, 23 (16.7%) from the Eastern Mediterranean region, 31 (22.5%) from the European region, nine (6.5%) from the South-East Asian region, and 22 (15.9%) from the Western Pacific region (Additional file 1: Table S2).

**Fig. 1:**
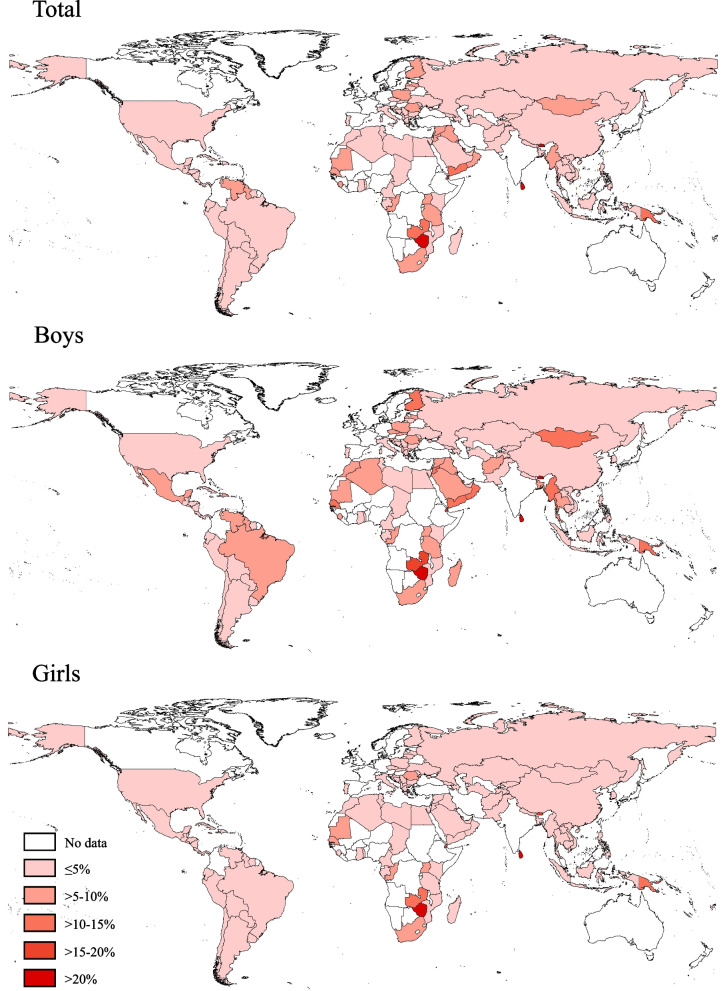
Prevalence of current smokeless tobacco use among adolescents aged 12–16 years in 138 countries/territories in 2010–2019

Based on the latest data from 138 countries in 2010-2019, the overall prevalence of current smokeless tobacco use was 4.4% (95% CI 4.0–4.9), with 5.7% (5.1–6.3) for boys, 3.1% (2.6–3.5) for girls, 3.9% (3.5–4.4) for adolescents aged 12–14 years, and 5.4% (4.8–5.9) for those aged 15–16 years (Table 1). The prevalence varied significantly across all 138 countries (from 0.0% in Tokelau to 51.6% in Kiribati), and by sex and age group within each of most countries (Fig. 1, and Additional file 1: Fig. S2 and Table S3). The prevalence was nearly five times higher among current cigarette smokers compared with non-smokers (14.3% vs. 3.0%), and nearly seven times higher among other tobacco product users compared with non-users (22.5% vs. 3.2%). The prevalence among adolescents whose both parents (9.8%), and mother only (5.6%) smoked was higher than those whose father only (4.4%) and neither parent (4.7%) smoked. The prevalence was highest in the South-East Asian region (6.1%), followed by the African region (5.4%), and lowest in the Western Pacific region (2.0%). The prevalence was highest in lower-middle-income countries (5.5%), followed by low-income countries (4.7%), and lowest in high-income countries (2.8%) (Table 1).

**Table 1:** Prevalence of current smokeless tobacco use among adolescents aged 12–16 years in 138 countries by sex, age group, cigarette smoking, other tobacco product use, parental smoking, WHO region, and World Bank income, 2010–2019

Group	No of countries	Prevalence of current smokeless tobacco use, % (95% CI)						
		Overall	Boys	Girls	*P*-value (boys vs. girls)	12–14 years	15–16 years	*P*-value (12–14 vs. 15–16 years)
**Total**	138	4.4 (4.0–4.9)	5.7 (5.1–6.3)	3.1 (2.6–3.5)	<0.0001^#^	3.9 (3.5–4.4)	5.4 (4.8–5.9)	<0.0001^#^
**Cigarette smoking**								
No	138	3.0 (2.6–3.5)	3.8 (3.2–4.4)	2.2 (1.8–2.7)	<0.0001^#^	2.8 (2.4–3.3)	3.5 (2.9–4.0)	0.0019
Yes	138	14.3 (12.6–16.1)	16.7 (14.6–18.8)	9.6 (7.7–11.4)	<0.0001^#^	13.1 (10.9–15.3)	15.7 (13.5–17.8)	0.059
*P*-value		<0.0001^#^	<0.0001^#^	<0.0001^#^		<0.0001^#^	<0.0001^#^	
**Other tobacco product use**								
No	132	3.2 (2.8–3.7)	4.1 (3.5–4.8)	2.4 (1.9–2.9)	<0.0001^#^	2.9 (2.5–3.4)	3.9 (3.2–4.5)	<0.0001^#^
Yes	132	22.5 (20.1–24.8)	23.7 (20.6–26.7)	20.0 (17.0–23.0)	0.077	21.7 (18.6–24.7)	23.4 (20.3–26.5)	0.38
*P*-value		<0.0001^#^	<0.0001^#^	<0.0001^#^		<0.0001^#^	<0.0001^#^	
**Parental smoking**								
Neither	87	4.7 (3.7–5.6)	5.8 (4.5–7.0)	3.6 (2.6–4.6)	0.0002^#^	4.2 (3.2–5.2)	5.5 (4.4–6.7)	0.0008
Father only	87	4.4 (3.9–4.9)	5.9 (5.1–6.7)	3.0 (2.4–3.6)	<0.0001^#^	3.8 (3.2–4.3)	5.9 (5.0–6.9)	<0.0001^#^
Mother only	87	5.6 (4.4–6.7)	8.0 (5.9–10.1)	3.3 (2.2–4.3)	<0.0001^#^	4.8 (3.4–6.2)	6.9 (5.0–8.7)	0.043
Both	87	9.8 (8.4–11.2)	11.6 (9.5–13.7)	8.1 (6.3–9.9)	0.012	9.7 (7.9–11.5)	10.0 (7.9–12.1)	0.84
*P*-value		<0.0001^#^	<0.0001^#^	<0.0001^#^		<0.0001^#^	<0.0001^#^	
**WHO region**								
Africa	23	5.4 (4.7–6.2)	6.5 (5.5–7.6)	4.3 (3.7–5.0)	<0.0001^#^	4.9 (4.1–5.8)	6.1 (5.3–6.9)	0.0038
Americas	30	3.2 (2.8–3.6)	4.4 (3.8–5.0)	2.0 (1.6–2.4)	<0.0001^#^	3.0 (2.6–3.4)	3.6 (3.0–4.3)	0.056
Eastern Mediterranean	23	4.8 (4.1–5.4)	6.7 (5.7–7.8)	2.7 (2.1–3.2)	<0.0001^#^	4.5 (3.6–5.3)	5.3 (4.4–6.3)	0.15
Europe	31	2.7 (2.3–3.0)	3.4 (2.8–3.9)	1.9 (1.5–2.3)	0.0001^#^	2.1 (1.8–2.5)	3.6 (3.1–4.2)	<0.0001^#^
South–East Asia	9	6.1 (4.4–7.7)	7.4 (5.3–9.4)	4.5 (2.8–6.3)	0.0089	5.0 (3.6–6.5)	9.2 (6.8–11.7)	<0.0001^#^
Western Pacific	22	2.0 (1.7–2.4)	2.4 (1.9–2.8)	1.7 (1.3–2.1)	0.0012	1.8 (1.4–2.2)	2.5 (1.9–3.0)	0.028
*P*-value		<0.0001^#^	<0.0001^#^	<0.0001^#^		<0.0001^#^	<0.0001^#^	
**World Bank income**								
Low income	20	4.7 (4.1–5.4)	5.3 (4.2–6.5)	4.0 (3.3–4.8)	0.064	4.4 (3.7–5.1)	5.4 (4.3–6.5)	0.082
Lower-middle income	40	5.5 (4.3–6.7)	7.1 (5.6–8.6)	3.8 (2.6–5.0)	<0.0001^#^	5.0 (3.8–6.2)	6.4 (5.0–7.9)	0.0084
Upper-middle income	45	3.8 (3.4–4.2)	5.3 (4.7–5.9)	2.3 (1.9–2.7)	<0.0001^#^	3.3 (2.9–3.7)	4.8 (4.1–5.5)	<0.0001^#^
High income	33	2.8 (2.4–3.1)	3.8 (3.3–4.4)	1.6 (1.3–1.9)	<0.0001^#^	1.9 (1.7–2.2)	4.2 (3.6–4.8)	<0.0001^#^
*P*-value		<0.0001^#^	0.0003^#^	<0.0001^#^		<0.0001^#^	0.017	

Prevalence estimates are weighte

*WHO*, World Health Organization

^#^ *P* value <0.00079 (0.05/63, which is Bonferroni’s corrected critical significance level)

In our analysis, at Bonferroni’s corrected significant level as 0.0031 (0.05/16), cigarette smoking (OR=2.00, 95% CI=1.68–2.39), other tobacco product use (OR=6.03, 95% CI=4.92–7.40), tobacco advertisement exposure (OR=1.44, 95% CI=1.19–1.74), and being offered free tobacco products (OR=2.01, 95% CI=1.66–2.42), not being taught about dangers of smoking (OR=1.28, 95% CI=1.09–1.50) were all positively associated with current smokeless tobacco use (Table 2).

**Table 2:** Factors associated with current smokeless tobacco use among adolescents aged 12–16 years based on the latest available data in 138 countries from 2010 to 2019

Variable	Prevalence (%)	*β*	OR (95% CI)	*P*-value
**Sex**				
Girls	3.1		1.00	
Boys	5.7	0.230	1.26 (1.03–1.53)	0.024
**Age group**				
12–14 years	3.9		1.00	
15–16 years	5.4	0.167	1.18 (1.01–1.38)	0.035
**Cigarette smoking**				
No	3.0		1.00	
Yes	14.3	0.694	2.00 (1.68–2.39)	<0.0001^#^
**Other tobacco product use**				
No	3.2		1.00	
Yes	22.5	1.797	6.03 (4.92–7.40)	<0.0001^#^
**Parental smoking status**				
Neither	4.7		1.00	
Father only	4.4	−0.124	0.88 (0.71–1.09)	0.25
Mother only	5.6	−0.155	0.86 (0.75–1.30)	0.47
Both	9.8	0.335	1.40 (1.08–1.82)	0.012
**Smoking status of closest friends**				
None	4.0		1.00	
Some	5.2	0.051	1.05 (0.83–1.33)	0.67
Most	7.6	0.090	1.09 (0.82–1.46)	0.54
All	15.2	0.431	1.54 (1.04–2.28)	0.031
**Tobacco advertisement exposure**				
No	3.4		1.00	
Yes	4.9	0.363	1.44 (1.19–1.74)	0.0002^#^
**Being offered free tobacco products**				
No	3.6		1.00	
Yes	12.1	0.696	2.01 (1.66–2.42)	<0.0001^#^
**Being taught about dangers of smoking**				
Yes	4.1		1.00	
No	4.8	0.245	1.28 (1.09–1.50)	0.0024^#^
**World Bank income**				
Low income	4.7		1.00	
Lower-middle income	5.5	0.027	1.03 (0.65–1.62)	0.91
Upper-middle income	3.8	−0.095	0.91 (0.73–1.13)	0.40
High income	2.8	−0.657	0.52 (0.40–0.68)	<0.0001^#^

*OR*, odds ratio; *CI*, confidence interval

Prevalence estimates are weighted

All variables listed in the table were introduced into logistic regression models.

^#^ *P* value <0.0031 (0.05/16, which is Bonferroni’s corrected critical significance level)

A total of 1,039,249 adolescents aged 12–16 years from 100 countries that had conducted two or more surveys between 1999 and 2019 were used to estimate the trend in the prevalence of smokeless tobacco use. From January 1999 to December 2019, 66 of 100 countries conducted two surveys, 23 conducted three surveys, 10 conducted four surveys, and one conducted 15 surveys; 83 countries conducted the surveys at the national level, and 17 countries conducted the surveys at the regional or subregional level (Additional file 1: Table S4).

**Fig. 2:**
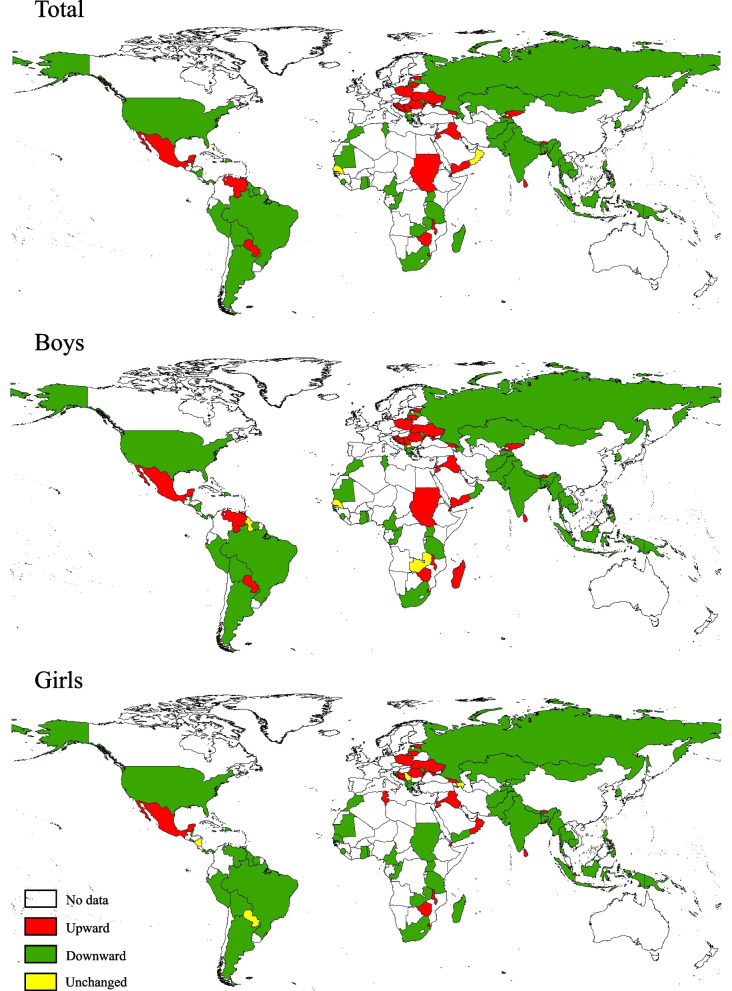
Secular trends in current smokeless tobacco use among adolescents aged 12–16 years in 100 countries/territories from 1999 to 2019

Secular trends in the prevalence of smokeless tobacco use differed significantly across all 100 countries from 1999 to 2019 (Fig. 2, and Additional file 1: Fig. S3 and Table S4). The prevalence of smokeless tobacco use decreased in 57 countries, increased in 32 countries, and remained unchanged in 11 countries. The prevalence substantially decreased over time in four WHO regions including Africa, Americas, South-East Asia, and Western Pacific. The decrease in prevalence among low-, lower-middle-, and upper-middle-income countries, was larger than in high-income countries. The results were similar by sex and age group (Table 3). However, the overall global secular trend estimates in the prevalence of smokeless tobacco use in 1999–2019 per 5 calendar years remained unchanged (*P*-value of linear trend > 0.0005 (0.05/100 using the Bonferroni’s correction), but decreased in some subgroups (e.g., the American region) (*P* < 0.0005) (Additional file 1: Table S5).

**Table 3:** Proportions of countries/territories with upward, downward, and unchanged trends in current smokeless tobacco use among adolescents aged 12–16 years in 100 countries from 1999 to 2019

Group	No. of countries	Proportions of countries/territories with different trends in current smokeless tobacco use (%)														
		Overall			Boys			Girls			12–14 years			15–16 years		
		Downward^#^	Upward^#^	Unchanged^†^	Downward^#^	Upward^#^	Unchanged^†^	Downward^#^	Upward^#^	Unchanged^†^	Downward^#^	Upward^#^	Unchanged^†^	Downward^#^	Upward^#^	Unchanged^†^
**Total**	100	57.0	32.0	11.0	55.0	33.0	12.0	61.0	23.0	16.0	59.0	26.0	15.0	49.0	39.0	12.0
**WHO region**																
Africa	17	70.6	17.6	11.8	64.7	23.5	11.8	76.5	17.6	5.9	64.7	17.6	17.6	70.6	23.5	5.9
Americas	23	73.9	13.0	13.0	69.6	13.0	17.4	78.3	4.3	17.4	78.3	4.3	17.4	56.5	30.4	13.0
Eastern Mediterranean	16	31.3	56.3	12.5	37.5	62.5	0.0	50.0	43.8	6.3	37.5	50.0	12.5	37.5	62.5	0.0
Europe	24	41.7	50.0	8.3	37.5	50.0	12.5	41.7	29.2	29.2	45.8	41.7	12.5	37.5	45.8	16.7
South-East Asia	10	70.0	30.0	0.0	70.0	30.0	0.0	70.0	30.0	0.0	70.0	30.0	0.0	50.0	50.0	0.0
Western Pacific	10	60.0	20.0	20.0	60.0	10.0	30.0	50.0	20.0	30.0	60.0	10.0	30.0	40.0	20.0	40.0
**World Bank income**																
Low income	16	62.5	37.5	0.0	56.3	43.8	0.0	68.8	25.0	6.3	50.0	37.5	12.5	56.3	43.8	0.0
Lower-middle income	32	62.5	28.1	9.4	62.5	31.3	6.3	71.9	21.9	6.3	65.6	25.0	9.4	59.4	37.5	3.1
Upper-middle income	32	53.1	31.3	15.6	46.9	31.3	21.9	53.1	21.9	25.0	56.3	25.0	18.8	43.8	40.6	15.6
High income	20	50.0	35.0	15.0	55.0	30.0	15.0	50.0	25.0	25.0	60.0	20.0	20.0	35.0	35.0	30.0

Data are presented as %

*WHO*, World Health Organization

Chi-square trend test was used to examine the trend in prevalence across all survey years (detailed results are listed in Table S4; Bonferroni’s corrected critical significance level is set at 0.0005 (0.05/100))

^#^ *P* for trend <0.0005; ^†^*P* for trend >0.0005)

## Discussion

In this study, the prevalence of current smokeless tobacco use was 4.4% among adolescents aged 12–16 years based on the latest data from 138 countries in 2010–2019. The prevalence was higher among boys than girls, among adolescents aged 15–16 years than those aged 12–14 years, and highest in the South-East Asian region but lowest in the Western Pacific region. Although the prevalence of smokeless tobacco use decreased in 57 of 100 countries, the prevalence increased or remained unchanged in 43 countries. Cigarette smoking, other tobacco product use, tobacco advertisement exposure, being offered free tobacco products, and not being taught about dangers of smoking were positively associated with smokeless tobacco use among adolescents.

The prevalence of current smokeless tobacco use varied largely between countries and WHO regions, with the lowest in Tokelau (0.0%) and highest in Kiribati (51.6%), and the lowest in the Western Pacific region (2.0%) and highest in the South-East Asian region (6.1%). However, Cahn et al. found that the prevalence of current smokeless tobacco use among youth aged 13–15 years was lowest in the European region (2.0%) and highest in the African region (7.45%) based on the GYTS data from 2007 to 2016 [8], which is different from our finding. It has been shown that smokeless tobacco is the dominant form of tobacco consumption in South-East Asia where over 80% of smokeless tobacco users live [6, 31]. The high social acceptance, low price, and easy availability make smokeless tobacco accessible and affordable among adolescents in this region [19]. The Western Pacific region is the only WHO region to achieve a 100% ratification rate for the FCTC [19] and most countries in this region have banned smokeless tobacco products [10], which may account for the low prevalence in this region. Our findings highlight the importance of strengthening implementation policy on smokeless tobacco control, especially in countries from the South-East Asian region.

Among 100 included countries, the prevalence of current smokeless tobacco use decreased in 57 and increased or leveled off in 43 countries from 1999 to 2019. We also found that the greatest decrease was observed in Timor-Leste (absolute change/5 years was − 42.9%) and the greatest increase was in Zimbabwe (21.8%). Our understanding is that no previous study has assessed the global secular trend of smokeless tobacco use among adolescents. In our study, the decreased trend in most countries might reflect the transition from using traditional tobacco products to using emerging tobacco products such as e-cigarettes and hookah [2–4], and effective implementation of smokeless tobacco control strategies and measures in those countries [21]. However, there were still 43 countries showing an increased or unchanged trend in the prevalence of smokeless tobacco use. This might be due to cigarette product restriction in some countries that shifts adolescents toward smokeless tobacco as an alternative for nicotine dependence [1]. Other factors might include sale of smokeless tobacco products to minors [20], weak enforcement and regulation, management difficulties in diverse products, marketing and sponsoring, lower taxes and prices, and deep-rooted social acceptance [9, 19, 21]. These findings suggest that demand-reduction measures for smokeless tobacco are necessary in countries with static or increased prevalence, such as bans on sale to minors, tax or price increases, pictorial warnings on packaging, and tailor-made education [20].

We found the prevalence of smokeless tobacco use was nearly five times as high among cigarette smokers and nearly seven times as high among other tobacco product users compared with their counterparts. Although our cross-sectional design limits our ability to draw causal inference from our findings, previous studies have found an increased likelihood of cigarette smoking and multiple tobacco use among exclusive smokeless tobacco users, but switching from smoke products to only smokeless tobacco use was less investigated [25, 26]. In addition, adolescents who used multiple tobacco products at the same time had a higher likelihood of developing nicotine addiction and continuing to use tobacco during adulthood [11, 16]. These findings highlight that timely prevention or intervention of smokeless tobacco use at an early age likely has important public health implications for reducing the prevalence of multiple tobacco use.

Adolescents exposed to tobacco-related advertising and sponsorship were more likely to start using smokeless tobacco. According to Article 13 Guidelines of the FCTC, all forms of tobacco advertising, promotion, and sponsorship should be prohibited. Despite the early achievements by some countries since implementing these proposals, more effort is needed to reduce tobacco-related marketing exposure among adolescents [20]. Notably, adolescents who had been taught about dangers of smoking in class were less likely to use smokeless tobacco, which underlines the importance of anti-tobacco activities that disseminate information about dangers of smoking to prevent youth smoking [32]. The prevalence among adolescents was higher in low- and lower-middle-income countries, which was shown previously [18]. This might be a result of smokeless tobacco products being more readily available and affordable to adolescents, and limited resources invested in smokeless tobacco control, in these countries [18]. The aforementioned findings highlight the importance of banning tobacco advertising and creating a smoke-free environment to protect youth — with more attention needed to address smokeless tobacco use among youth in low and lower-middle-income countries.

Strengths of this study were the use of the latest global data to assess the prevalence of smokeless tobacco use among adolescents and we are the first to determine secular trends in the prevalence. Moreover, data were from the global surveillance instrument with the same sampling frame and standardized questions, which allow direct comparisons across countries. However, several limitations should be considered. First, information on smokeless tobacco use was self-reported, and recall bias might influence the results. However, a previous study reported a good test-retest reliability of the GYTS questionnaire [33]. Second, the GYTS data do not provide information on smokeless tobacco use by different types, which should be considered in future surveys. Third, current smokeless tobacco use was defined based on response to whether or not the participants used smokeless tobacco products during the past 30 days, rather than based on the use intensity or frequency, making it difficult to distinguish between experimental and regular users. Fourth, as our study only included adolescents aged 12–16 years in school, our findings might have limited generalizability to adolescents of other age groups and those out of school. Fifth, the regional generalizability of the results was limited given that in some regions, such as Europe, data were missing for a great number of countries that might influence the findings. Sixth, we used data from a wide range of survey years (2010–2019) to estimate the prevalence of current smokeless tobacco use, which might influence the pooled prevalence. However, 81.2% (112/138) of included countries conducted the surveys in 2013–2018. The pooled prevalence based on 112 countries in 2013–2018 was 4.5% (95% CI 3.9–5.1), which was largely similar to the pooled prevalence based on 138 countries in 2010–2019 (4.4%, 95% CI 4.0–4.9). Seventh, only 100 out of 138 countries provided sufficient data for a trend analysis between 1999 and 2018. Eighth, no causal inference can be made between associated factors and smokeless tobacco use due to the cross-sectional design.

## Conclusions

Smokeless tobacco use among adolescents remains a public health issue worldwide. Although the prevalence among adolescents decreased from 1999 to 2019 in most countries, it remains high especially in the South-East Asian region. Our findings emphasize full implementation and enforcement of the WHO FCTC provisions on smokeless tobacco, which is integral to stemming the smokeless tobacco epidemic among adolescents.

## Supplementary Information

**Additional file 1: Table S1.** The codes for dependent and independent variables included in multivariable logistic regression models. **Table S2.** Characteristics of Global Youth Tobacco Surveys among adolescents aged 12-16 years in 138 countries/territories in 2010-2019. **Table S3.** Prevalence of current smokeless tobacco use among adolescents aged 12-16 years in 138 countries by sex, age group, and country/territory, 2010-2019. **Table S4.** Trends in the prevalence of current smokeless tobacco use among adolescents aged 12-16 years in 100 countries from 1999 to 2019 by country/territory. **Table S5.** Linear trends per 5 calendar years in the prevalence of current smokeless tobacco use among adolescents aged 12-16 years in 100 countries from 1999 to 2019. **Figure S1.** Flow chart of the inclusion/exclusion of countries/territories. **Figure S2.** Prevalence of current smokeless tobacco use among adolescents aged 12-16 years by age group in 138 countries/territories in 2010-2019. **Figure S3.** Secular trends in current smokeless tobacco use among adolescents aged 12-16 years by age group in 100 countries/territories from 1999 to 2019.

## Data Availability

The datasets generated and/or analyzed during the current study are publicly available in the the U.S. Centers for Disease Control and Prevention website (https://www.cdc.gov/tobacco/global/gtss/gtssdata/index.html or https://www.cdc.gov/tobacco/data_statistics/surveys/nyts/index.htm).

## Abbreviations

WHO: World Health Organization
FCTC: Framework Convention on Tobacco Control
GYTS: Global Youth Tobacco Survey
NYTS: National Youth Tobacco Survey
U.S.: United States
OR: Odds ratio
CI: Confidence interval
