# Supplementary material for: Prevalence and trend of smokeless tobacco use and its associated factors among adolescents aged 12–16 years in 138 countries/territories, 1999–2019
Source: BMC Med. 2022 Nov 25;20:460. doi: 10.1186/s12916-022-02662-0 (PMC9700970; doi:10.1186/s12916-022-02662-0)
Supplement: Supplementary file 1 — Additional file 1: Table S1. The codes for dependent and independent variables included in multivariable logistic regression models. Table S2. Characteristics of Global Youth Tobacco Surveys among adolescents aged 12-16 years in 138 countries/territories in 2010-2019. Table S3. Prevalence of current smokeless tobacco use among adolescents aged 12-16 years in 138 countries by sex, age group, and country/territory, 2010-2019. Table S4. Trends in the prevalence of current smokeless tobacco use among adolescents aged 12-16 years in 100 countries from 1999 to 2019 by country/territory. Table S5. Linear trends per 5 calendar years in the prevalence of current smokeless tobacco use among adolescents aged 12-16 years in 100 countries from 1999 to 2019. Figure S1. Flow chart of the inclusion/exclusion of countries/territories. Figure S2. Prevalence of current smokeless tobacco use among adolescents aged 12-16 years by age group in 138 countries/territories in 2010-2019. Figure S3. Secular trends in current smokeless tobacco use among adolescents aged 12-16 years by age group in 100 countries/territories from 1999 to 2019. [file 12916_2022_2662_MOESM1_ESM.doc]

**Table S1. The codes for dependent and independent variables included in multivariable logistic regression model**

| **Variable** | **Code** |  | **Variable** | **Code** |
| --- | --- | --- | --- | --- |
| Current smokeless tobacco use |  |  | Smoking status of closest friends |  |
| No | 0 |  | None | 0 |
| Yes | 1 |  | Some | 1 |
| Sex |  |  | Most | 2 |
| Girls | 0 |  | All | 3 |
| Boys | 1 |  | Tobacco advertisement exposure |  |
| Age group |  |  | No | 0 |
| 12-14 years | 0 |  | Yes | 1 |
| 15-16 years | 1 |  | Being offered free tobacco products |  |
| Cigarette smoking |  |  | No | 0 |
| No | 0 |  | Yes | 1 |
| Yes | 1 |  | Being taught about dangers of smoking |  |
| Other tobacco product use |  |  | Yes | 0 |
| No | 0 |  | No | 1 |
| Yes | 1 |  | World Bank income |  |
| Parental smoking status |  |  | Low income | 0 |
| Neither | 0 |  | Lower-Middle income | 1 |
| Father only | 1 |  | Upper-Middle income | 2 |
| Mother only | 2 |  | High income | 3 |
| Both | 3 |  |  |  |

**Table S2. Characteristics of Global Youth Tobacco Surveys among adolescents aged 12-16 years in 138 countries/territories in 2010-19**

| **Country/territory** | **Representativeness** | **Survey year** | **Sample size** | **Boys, %** |
| --- | --- | --- | --- | --- |
| **Africa** |  |  |  |  |
| Algeria | National | 2013 | 5226 | 44.9 |
| Cameroon | National | 2014 | 2351 | 52.3 |
| Chad | National | 2019 | 1441 | 60.9 |
| Comoros | National | 2015 | 2225 | 46.4 |
| Congo | National | 2019 | 5031 | 50.3 |
| Gabon | National | 2014 | 1187 | 46.5 |
| Gambia | National | 2017 | 9169 | 43.9 |
| Ghana | National | 2017 | 5193 | 50.6 |
| Kenya | National | 2013 | 1690 | 50.3 |
| Madagascar | National | 2018 | 2196 | 47.7 |
| Mauritania | National | 2018 | 2979 | 51.5 |
| Mauritius | National | 2016 | 3841 | 48.3 |
| Mozambique | National | 2013 | 4048 | 49.7 |
| Sao Tome and Principe | National | 2010 | 5375 | 45.2 |
| Senegal | National | 2013 | 1227 | 54.2 |
| Seychelles | National | 2015 | 2327 | 49.3 |
| Sierra Leone | National | 2017 | 4335 | 47.8 |
| South Africa | National | 2011 | 6159 | 45.0 |
| Togo | National | 2019 | 3176 | 55.2 |
| Uganda | National | 2018 | 2888 | 47.6 |
| United Republic of Tanzania | National | 2016 | 3519 | 48.6 |
| Zambia | National | 2011 | 2661 | 47.3 |
| Zimbabwe | National | 2014 | 5948 | 49.0 |
| **Americas** |  |  |  |  |
| Antigua and Barbuda | National | 2017 | 1941 | 50.9 |
| Argentina | National | 2018 | 1353 | 49.5 |
| Bahamas | National | 2013 | 1257 | 55.4 |
| Barbados | National | 2013 | 1644 | 51.4 |
| Belize | National | 2014 | 1675 | 48.8 |
| Bolivia | National | 2018 | 4456 | 50.3 |
| Brazil | Subnational | 2012 | 1372 | 46.0 |
| Chile | Subnational | 2016 | 10727 | 49.3 |
| Costa Rica | National | 2013 | 2753 | 50.1 |
| Cuba | National | 2018 | 3943 | 50.6 |
| Dominican Republic | National | 2016 | 1227 | 47.2 |
| Ecuador | National | 2016 | 5071 | 49.9 |
| El Salvador | National | 2015 | 2889 | 50.2 |
| Grenada | National | 2016 | 1965 | 49.8 |
| Guatemala | National | 2015 | 3851 | 52.1 |
| Guyana | National | 2015 | 1541 | 49.8 |
| Honduras | National | 2016 | 3233 | 47.3 |
| Jamaica | National | 2017 | 1346 | 44.1 |
| Mexico | National | 2011 | 2422 | 47.9 |
| Nicaragua | National | 2019 | 7446 | 50.0 |
| Panama | National | 2017 | 2464 | 49.9 |
| Paraguay | National | 2019 | 4486 | 50.0 |
| Peru | National | 2019 | 3814 | 50.1 |
| Saint Lucia | National | 2017 | 1444 | 50.9 |
| Saint Vincent and the Grenadines | National | 2018 | 1289 | 49.7 |
| Suriname | National | 2016 | 1767 | 47.8 |
| Trinidad and Tobago | National | 2017 | 3384 | 48.2 |
| United States of America ^*^ | National | 2019 | 13689 | 52.2 |
| Uruguay | National | 2014 | 4414 | 46.3 |
| Venezuela | National | 2010 | 2231 | 50.3 |
| **Eastern Mediterranean** |  |  |  |  |
| Afghanistan | Subnational | 2017 | 1439 | 57.1 |
| Bahrain | National | 2015 | 3243 | 49.8 |
| Djibouti | National | 2013 | 1622 | 54.6 |
| Egypt | National | 2014 | 2259 | 52.3 |
| Gaza Strip | Subnational | 2019 | 1402 | 47.2 |
| Iraq | National | 2019 | 2051 | 55.0 |
| Jordan | National | 2014 | 2101 | 50.4 |
| Kuwait | National | 2016 | 2370 | 47.9 |
| Libyan Arab Jamahiriya | National | 2010 | 1756 | 48.0 |
| Morocco | National | 2016 | 3706 | 43.7 |
| Oman | National | 2016 | 2013 | 49.1 |
| Pakistan | National | 2013 | 7552 | 59.2 |
| Qatar | National | 2018 | 1973 | 47.3 |
| Saudi Arabia | National | 2010 | 2214 | 50.4 |
| Syrian Arab Republic | National | 2010 | 1597 | 48.3 |
| Tunisia | National | 2017 | 2368 | 50.0 |
| United Arab Emirates | National | 2013 | 3877 | 51.9 |
| UNRWA GAZA (Palestine) ^a^ | Regional | 2013 | 1665 | 50.3 |
| UNRWA Jordan | Regional | 2014 | 1315 | 51.4 |
| UNRWA Lebanon | Regional | 2013 | 1394 | 45.1 |
| UNRWA West bank (Palestine) ^a^ | Regional | 2014 | 1331 | 40.7 |
| West BANK | Regional | 2016 | 1405 | 48.5 |
| Yemen | National | 2014 | 1863 | 60.4 |
| **Europe** |  |  |  |  |
| Albania | National | 2015 | 4483 | 52.7 |
| Azerbaijan | National | 2016 | 2158 | 53.9 |
| Belarus | National | 2015 | 2912 | 49.4 |
| Bosnia and Herzegovina | National | 2013 | 11286 | 51.6 |
| Bulgaria | National | 2015 | 3850 | 51.8 |
| Croatia (Hrvatska) | National | 2016 | 3168 | 49.6 |
| Czech Republic | National | 2016 | 3890 | 51.4 |
| Finland | National | 2012 | 4777 | 50.7 |
| Georgia | National | 2017 | 1238 | 50.4 |
| Greece | National | 2013 | 4506 | 51.6 |
| Italy | National | 2018 | 1621 | 52.0 |
| Kazakhstan | National | 2014 | 2017 | 49.6 |
| Kosovo | National | 2016 | 4933 | 51.5 |
| Kyrgyzstan | National | 2019 | 6041 | 50.7 |
| Latvia | National | 2019 | 4112 | 51.1 |
| Lithuania | National | 2018 | 2928 | 50.6 |
| Macedonia | National | 2016 | 4962 | 52.3 |
| Malta | National | 2017 | 1237 | 55.5 |
| Montenegro | National | 2018 | 4036 | 49.3 |
| Poland | National | 2016 | 4933 | 50.9 |
| Portugal | National | 2013 | 10246 | 51.4 |
| Republic of Moldova | National | 2019 | 4544 | 50.7 |
| Romania | National | 2017 | 5290 | 51.3 |
| Russian Federation | Subnational | 2015 | 6487 | 50.6 |
| San Marino | National | 2018 | 600 | 54.7 |
| Serbia | National | 2017 | 3757 | 49.5 |
| Slovakia | National | 2016 | 3917 | 49.9 |
| Slovenia | National | 2017 | 2490 | 51.5 |
| Tajikistan | National | 2019 | 3919 | 51.2 |
| Turkmenistan | National | 2015 | 8235 | 49.7 |
| Ukraine | National | 2017 | 3844 | 50.3 |
| **South-East Asia** |  |  |  |  |
| Bangladesh | National | 2013 | 3200 | 65.1 |
| Bhutan | National | 2013 | 1926 | 45.3 |
| Indonesia | National | 2014 | 5778 | 50.7 |
| Maldives | National | 2011 | 2132 | 53.6 |
| Myanmar | National | 2016 | 3464 | 45.7 |
| Nepal | National | 2011 | 2365 | 48.2 |
| Sri Lanka | National | 2015 | 1498 | 50.1 |
| Thailand | National | 2015 | 1849 | 50.9 |
| Timor-Leste | National | 2013 | 1839 | 51.8 |
| **Western Pacific** |  |  |  |  |
| Brunei Darussalam | National | 2013 | 1418 | 51.0 |
| Cambodia | National | 2016 | 2773 | 48.1 |
| China | National | 2013 | 155117 | 51.8 |
| Cook Islands | National | 2016 | 474 | 47.7 |
| Fiji | National | 2016 | 2317 | 48.8 |
| Guam (United States) ^a^ | National | 2017 | 1713 | 52.4 |
| Kiribati | National | 2018 | 1876 | 47.7 |
| Laos | National | 2016 | 5630 | 50.3 |
| Macao (China) ^a^ | Regional | 2015 | 1626 | 50.2 |
| Marshall Islands | National | 2016 | 2240 | 44.1 |
| Micronesia | National | 2013 | 3539 | 46.0 |
| Mongolia | National | 2014 | 6994 | 48.4 |
| Northern Mariana Islands | National | 2014 | 2075 | 50.8 |
| Palau | National | 2017 | 1053 | 49.1 |
| Papua New Guinea | National | 2016 | 1744 | 50.8 |
| Philippines | National | 2015 | 7649 | 48.1 |
| Samoa | National | 2017 | 1420 | 48.0 |
| South Korea | National | 2013 | 4153 | 51.8 |
| Tokelau | National | 2014 | 94 | 51.3 |
| Tonga | National | 2010 | 1952 | 42.4 |
| Tuvalu | National | 2018 | 585 | 44.1 |
| Vanuatu | National | 2017 | 1569 | 47.0 |
| Viet Nam | National | 2014 | 3527 | 49.7 |
| Total | — | — | 605808 | 51.4 |

UNRWA: United Nations Relief and Works Agency.

^a^ Macao is the special administrative region of China; Guam is an overseas territory of the United States; Gaza and West bank are territories of Palestine.^*^ Data in the U.S. were from the National Youth Tobacco Survey, which is described in the main text.

**Table S3. Prevalence of current smokeless tobacco use among adolescents aged 12-16 years in 138 countries by sex, age group, and country/territory, 2010-19**

| Country/territory | Prevalence of current smokeless tobacco use, % (95% CI) | | | | | | | | |
| --- | --- | --- | --- | --- | --- | --- | --- | --- | --- |
|  | Overall | Boys | Girls | *P*-value (boys vs. girls) | 12-14 years | 15-16 years | | *P*-value (12-14 vs. 15-16 years) | |
| **Africa** |  |  |  |  |  |  | |  | |
| Algeria | 3.9 (3.0-4.8) | 8.4 (6.2-10.5) | 0.3 (0.0-0.5) | <0.0001^#^ | 2.0 (1.2-2.7) | 6.1 (4.9-7.3) | | <0.0001^#^ | |
| Cameroon | 3.6 (2.3-4.9) | 4.7 (3.0-6.4) | 2.5 (1.4-3.5) | 0.0002 | 3.4 (2.3-4.5) | 4.1 (2.0-6.1) | | 0.41 | |
| Chad | 3.8 (2.8-4.8) | 3.4 (2.2-4.6) | 4.4 (2.7-6.1) | 0.32 | 4.5 (2.7-6.2) | 3.4 (2.2-4.6) | | 0.33 | |
| Comoros | 8.1 (6.5-9.7) | 10.9 (8.6-13.1) | 5.7 (4.1-7.3) | <0.0001^#^ | 7.7 (5.6-9.9) | 8.4 (6.7-10.2) | | 0.57 | |
| Congo | 6.9 (4.9-8.9) | 8.1 (5.7-10.6) | 5.6 (3.6-7.6) | 0.0098 | 6.9 (4.6-9.2) | 6.8 (4.9-8.7) | | 0.89 | |
| Gabon | 3.8 (2.0-5.6) | 3.3 (0.6-6.1) | 4.2 (2.3-6.1) | 0.60 | 3.0 (0.8-5.3) | 4.8 (3.7-6.8) | | 0.22 | |
| Gambia | 1.7 (1.3-2.0) | 2.6 (1.9-3.4) | 0.9 (0.6-1.2) | <0.0001^#^ | 1.6 (1.2-2.1) | 1.7 (1.1-2.3) | | 0.74 | |
| Ghana | 3.4 (2.3-4.5) | 3.0 (2.0-4.0) | 3.9 (2.1-5.7) | 0.30 | 2.9 (1.6-4.2) | 4.1 (2.6-5.5) | | 0.15 | |
| Kenya | 4.2 (3.4-5.0) | 5.0 (3.4-6.6) | 3.4 (2.5-4.4) | 0.14 | 3.9 (3.2-4.6) | 4.7 (3.0-6.4) | | 0.33 | |
| Madagascar | 4.2 (2.6-5.8) | 5.7 (3.0-8.4) | 2.8 (1.2-4.3) | 0.036 | 3.6 (1.5-5.7) | 5.0 (2.3-7.7) | | 0.43 | |
| Mauritania | 6.7 (5.7-7.7) | 6.6 (4.9-8.3) | 6.7 (5.1-8.3) | 0.94 | 6.6 (5.5-7.6) | 6.8 (5.1-8.5) | | 0.75 | |
| Mauritius | 2.5 (2.0-3.0) | 2.6 (1.7-3.4) | 2.5 (1.5-3.5) | 0.93 | 2.1 (1.4-2.7) | 3.6 (2.1-5.2) | | 0.073 | |
| Mozambique | 4.2 (3.4-5.0) | 4.7 (3.6-5.9) | 3.7 (2.8-4.6) | 0.12 | 4.4 (3.2-5.7) | 4.0 (3.1-4.9) | | 0.63 | |
| Sao Tome and Principe | 23.4 (22.2-24.5) | 27.1 (25.3-28.8) | 20.3 (18.9-21.8) | <0.0001^#^ | 22.1 (20.4-23.7) | 24.4 (22.9-26.0) | | 0.045 | |
| Senegal | 8.3 (3.6-13.0) | 10.6 (4.1-17.1) | 5.6 (2.1-9.1) | 0.011 | 7.9 (2.8-13.1) | 8.8 (4.3-13.3) | | 0.51 | |
| Seychelles | 1.8 (1.1-2.4) | 2.8 (1.6-4.0) | 0.8 (0.3-1.3) | 0.0002 | 1.9 (1.0-2.8) | 1.6 (0.7-2.5) | | 0.67 | |
| Sierra Leone | 6.3 (2.9-9.6) | 8.0 (3.2-12.8) | 4.7 (1.6-7.7) | 0.098 | 7.5 (1.7-13.4) | 5.3 (1.7-8.9) | | 0.50 | |
| South Africa | 8.2 (6.4-10.0) | 9.8 (7.6-12.0) | 6.8 (5.1-8.6) | 0.0034 | 6.0 (4.9-7.0) | 9.4 (7.4-11.3) | | 0.0064 | |
| Togo | 2.0 (1.2-2.8) | 2.5 (1.5-3.4) | 1.5 (0.6-2.4) | 0.068 | 1.7 (0.8-2.5) | 2.7 (1.5-3.9) | | 0.048 | |
| Uganda | 6.2 (4.8-7.6) | 6.4 (4.5-8.4) | 6.1 (3.4-8.8) | 0.85 | 4.7 (3.8-5.6) | 7.5 (5.0-10.1) | | 0.020 | |
| United Republic of Tanzania | 5.1 (3.6-6.6) | 5.7 (3.8-7.6) | 4.5 (2.5-6.4) | 0.36 | 5.9 (4.2-7.6) | 3.6 (1.9-5.3) | 0.0070 | | |
| Zambia | 14.7 (12.1-17.2) | 15.4 (10.7-20.1) | 14.0 (13.4-14.6) | 0.30 | 13.6 (11.4-15.8) | 15.9 (14.3-17.5) | 0.093 | | |
| Zimbabwe | 31.4 (25.9-36.9) | 32.1 (27.3-36.8) | 30.7 (23.4-38.1) | 0.66 | 26.9 (22.3-31.6) | 40.6 (29.8-51.4) | 0.0050 | | |
| **Americas** |  |  |  |  |  |  |  | | |
| Antigua and Barbuda | 2.0 (1.3-2.8) | 2.4 (1.3-3.6) | 1.6 (0.8-2.4) | 0.21 | 1.6 (0.7-2.5) | 2.5 (1.4-3.6) | 0.18 | | |
| Argentina | 1.5 (0.8-2.2) | 2.3 (1.1-3.5) | 0.7 (0.1-1.4) | 0.015 | 1.5 (0.5-2.4) | 1.6 (0.6-2.6) | 0.84 | | |
| Bahamas | 2.3 (1.1-3.4) | 3.0 (1.4-4.6) | 1.4 (0.3-2.4) | 0.035 | 2.2 (1.0-3.4) | 2.6 (0.0-5.2) | 0.76 | | |
| Barbados | 3.1 (2.1-4.0) | 2.8 (1.7-3.9) | 3.3 (2.0-4.6) | 0.57 | 2.8 (1.9-3.6) | 4.8 (2.4-7.2) | 0.032 | | |
| Belize | 2.8 (1.9-3.7) | 3.4 (1.9-4.9) | 2.2 (1.4-3.1) | 0.14 | 1.8 (0.9-2.7) | 4.4 (2.5-6.2) | 0.025 | | |
| Bolivia | 3.1 (2.3-3.9) | 3.9 (2.7-5.1) | 2.3 (1.5-3.0) | 0.0027 | 2.6 (1.6-3.6) | 3.6 (2.6-4.6) | 0.12 | | |
| Brazil | 3.9 (2.5-5.3) | 5.2 (2.9-7.6) | 2.7 (1.5-3.9) | 0.017 | 3.9 (1.8-6.0) | 3.8 (1.8-5.8) | 0.94 | | |
| Chile | 1.3 (0.9-1.6) | 1.4 (0.9-1.9) | 1.1 (0.8-1.5) | 0.27 | 0.9 (0.6-1.3) | 1.7 (1.2-2.3) | 0.0097 | | |
| Costa Rica | 1.7 (1.1-2.3) | 1.8 (1.1-2.5) | 1.6 (0.9-2.3) | 0.52 | 1.7 (0.9-2.6) | 1.7 (1.0-2.3) | 0.87 | | |
| Cuba | 2.4 (1.3-3.5) | 2.2 (0.4-3.9) | 2.6 (1.3-3.8) | 0.72 | 1.5 (1.0-2.1) | 4.2 (1.3-7.2) | 0.0017 | | |
| Dominican Republic | 4.5 (2.7-6.2) | 7.6 (4.4-10.9) | 1.6 (0.3-2.9) | 0.0004 | 4.1 (1.6-6.5) | 4.6 (2.5-6.8) | 0.72 | | |
| Ecuador | 3.2 (2.4-3.9) | 4.0 (3.0-5.1) | 2.3 (1.3-3.2) | 0.016 | 2.9 (2.0-3.8) | 3.6 (2.7-4.4) | 0.21 | | |
| El Salvador | 2.0 (1.3-2.6) | 2.0 (1.1-3.0) | 1.9 (1.3-2.6) | 0.83 | 1.6 (0.7-2.4) | 2.5 (1.5-3.5) | 0.15 | | |
| Grenada | 1.6 (1.1-2.2) | 1.8 (1.0-2.7) | 1.4 (0.6-2.3) | 0.50 | 1.9 (1.1-2.7) | 1.3 (0.6-2.0) | 0.28 | | |
| Guatemala | 2.5 (1.9-3.2) | 3.3 (2.4-4.2) | 1.7 (1.0-2.4) | 0.0015 | 2.8 (1.9-3.8) | 2.1 (1.4-2.9) | 0.31 | | |
| Guyana | 6.6 (4.2-9.0) | 8.7 (4.3-13.2) | 4.5 (2.7-6.3) | 0.050 | 6.1 (3.6-8.6) | 12.9 (3.6-22.3) | 0.083 | | |
| Honduras | 2.0 (1.3-2.6) | 2.1 (1.2-3.0) | 1.8 (1.0-2.6) | 0.62 | 1.5 (0.8-2.3) | 3.8 (1.8-5.7) | 0.017 | | |
| Jamaica | 2.5 (1.7-3.4) | 2.9 (1.5-4.2) | 2.3 (1.2-3.3) | 0.49 | 2.5 (1.3-3.8) | 2.5 (1.4-3.6) | 0.97 | | |
| Mexico | 5.0 (4.0-6.1) | 5.8 (4.2-7.4) | 4.3 (3.0-5.7) | 0.15 | 4.8 (3.7-5.9) | 6.1 (3.4-8.8) | 0.31 | | |
| Nicaragua | 3.3 (2.8-3.9) | 3.8 (3.0-4.6) | 2.9 (2.2-3.6) | 0.093 | 2.8 (2.3-3.4) | 4.7 (3.4-5.9) | 0.0027 | | |
| Panama | 2.5 (1.8-3.2) | 2.4 (1.5-3.3) | 2.6 (1.6-3.6) | 0.81 | 2.3 (1.5-3.1) | 3.0 (1.6-4.4) | 0.35 | | |
| Paraguay | 1.7 (1.1-2.2) | 2.1 (1.1-3.1) | 1.2 (0.3-2.2) | 0.32 | 1.5 (1.0-2.0) | 2.8 (0.2-5.4) | 0.18 | | |
| Peru | 1.7 (1.3-2.1) | 2.1 (1.5-2.7) | 1.3 (0.6-2.0) | 0.14 | 1.9 (1.2-2.6) | 1.3 (0.8-1.9) | 0.29 | | |
| Saint Lucia | 7.1 (5.0-9.3) | 9.9 (6.7-13.2) | 4.3 (2.5-6.0) | <0.0001^#^ | 6.1 (4.0-8.2) | 10.8 (5.5-16.1) | 0.052 | | |
| Saint Vincent and the Grenadines | 5.8 (4.2-7.4) | 7.9 (5.2-10.5) | 3.8 (2.4-5.2) | 0.0011 | 4.7 (2.8-6.5) | 6.5 (4.3-8.7) | 0.23 | | |
| Suriname | 2.9 (1.9-3.9) | 4.5 (2.9-6.1) | 1.4 (0.6-2.2) | 0.0007 | 2.1 (1.0-3.2) | 3.8 (2.5-5.0) | 0.012 | | |
| Trinidad and Tobago | 7.2 (6.1-8.3) | 9.8 (8.3-11.3) | 4.8 (3.6-6.1) | <0.0001^#^ | 5.7 (4.3-7.1) | 9.5 (7.7-11.4) | 0.0002 | | |
| United States of America | 2.5 (1.9-3.1) | 3.9 (2.9-4.8) | 0.9 (0.5-1.3) | <0.0001^#^ | 1.7 (1.3-2.1) | 3.6 (2.4-4.9) | <0.0001^#^ | | |
| Uruguay | 3.1 (2.6-3.7) | 3.8 (2.9-4.8) | 2.5 (1.9-3.1) | 0.017 | 3.2 (2.6-3.9) | 2.9 (1.9-3.8) | 0.56 | | |
| Venezuela | 5.2 (4.3-6.1) | 7.8 (6.2-9.4) | 2.5 (1.6-3.4) | <0.0001^#^ | 4.8 (4.0-5.7) | 8.0 (5.0-11.0) | 0.031 | | |
| **Eastern Mediterranean** |  |  |  |  |  |  |  | | |
| Afghanistan | 4.4 (3.0-5.7) | 5.6 (3.4-7.8) | 2.7 (1.4-4.1) | 0.032 | 4.3 (2.8-5.9) | 4.5 (2.4-6.5) | 0.93 | | |
| Bahrain | 13.0 (8.5-17.5) | 20.3 (18.2-22.4) | 5.8 (4.1-7.5) | <0.0001^#^ | 10.0 (5.9-14.1) | 18.4 (11.5-25.4) | 0.0015 | | |
| Djibouti | 15.6 (12.6-18.6) | 17.5 (13.9-21.1) | 13.3 (9.6-17.1) | 0.080 | 13.8 (10.0-17.6) | 18.5 (14.8-22.2) | 0.079 | | |
| Egypt | 2.5 (0.8-4.1) | 3.8 (1.1-6.6) | 1.0 (0.1-1.9) | 0.0014 | 2.3 (0.3-4.3) | 2.8 (0.5-5.2) | 0.69 | | |
| Gaza Strip | 4.2 (1.8-6.7) | 6.2 (2.3-10.2) | 2.4 (0.9-3.9) | 0.0035 | 5.0 (1.9-8.1) | 2.7 (0.0-5.6) | 0.26 | | |
| Iraq | 5.9 (3.9-7.9) | 7.3 (4.8-9.9) | 4.1 (2.4-5.9) | 0.0034 | 5.0 (2.8-7.3) | 7.2 (4.0-10.4) | 0.20 | | |
| Jordan | 9.0 (5.7-12.3) | 13.5 (9.5-17.5) | 4.4 (3.1-5.6) | <0.0001^#^ | 7.6 (5.1-10.0) | 14.0 (7.4-20.7) | 0.0006 | | |
| Kuwait | 6.8 (5.1-8.4) | 9.6 (7.2-11.9) | 4.2 (3.1-5.4) | <0.0001^#^ | 4.7 (2.9-6.4) | 9.4 (6.7-12.0) | 0.0017 | | |
| Libyan Arab Jamahiriya | 2.7 (1.7-3.7) | 3.0 (1.7-4.2) | 2.4 (1.2-3.7) | 0.49 | 2.6 (1.4-3.7) | 3.0 (1.5-4.5) | 0.65 | | |
| Morocco | 3.8 (2.2-5.3) | 6.0 (3.3-8.7) | 2.1 (0.9-3.2) | 0.0002 | 3.0 (1.5-4.4) | 5.0 (2.9-7.0) | 0.0049 | | |
| Oman | 6.9 (4.0-9.8) | 11.8 (8.4-15.2) | 2.2 (1.3-3.1) | <0.0001^#^ | 4.9 (2.6-7.1) | 8.3 (4.4-12.1) | 0.015 | | |
| Pakistan | 3.7 (2.8-4.5) | 4.5 (4.2-4.7) | 2.5 (1.2-3.7) | 0.050 | 3.6 (2.5-4.8) | 3.7 (2.3-5.2) | 0.94 | | |
| Qatar | 13.0 (9.6-16.5) | 19.3 (14.3-24.3) | 7.5 (5.7-9.2) | <0.0001^#^ | 12.3 (8.2-16.3) | 15.4 (12.8-18.1) | 0.11 | | |
| Saudi Arabia | 3.8 (2.5-5.1) | 5.2 (2.9-7.4) | 2.3 (1.3-3.4) | 0.0085 | 2.9 (1.5-4.2) | 5.1 (3.1-7.0) | 0.028 | | |
| Syrian Arab Republic | 6.1 (4.2-8.0) | 8.0 (3.5-12.4) | 4.3 (2.4-6.2) | 0.15 | 6.7 (4.0-9.3) | 3.8 (1.6-5.9) | 0.19 | | |
| Tunisia | 2.5 (1.7-3.2) | 3.3 (2.2-4.4) | 1.6 (0.8-2.5) | 0.012 | 2.6 (1.8-3.4) | 2.1 (0.8-3.3) | 0.43 | | |
| United Arab Emirates | 3.6 (2.7-4.6) | 4.6 (3.0-6.2) | 2.6 (1.8-3.3) | 0.0070 | 3.1 (2.1-4.0) | 4.5 (2.9-6.1) | 0.078 | | |
| UNRWA GAZA (Palestine) ^a^ | 13.1 (9.2-17.0) | 16.4 (10.9-22.0) | 9.7 (5.8-13.7) | 0.028 | 12.9 (8.9-17.0) | 14.4 (10.5-18.2) | 0.40 | | |
| UNRWA Jordan | 13.7 (9.3-18.0) | 21.4 (17.4-25.5) | 5.5 (4.2-6.7) | <0.0001^#^ | 12.6 (8.9-16.3) | 17.3 (8.8-25.9) | 0.11 | | |
| UNRWA Lebanon | 12.5 (9.5-15.6) | 16.9 (12.8-20.9) | 9.0 (5.9-12.1) | <0.0001^#^ | 10.7 (7.6-13.8) | 17.1 (12.2-21.9) | 0.011 | | |
| UNRWA West bank (Palestine) ^a^ | 15.7 (11.5-19.9) | 23.9 (21.5-26.3) | 10.1 (7.2-12.9) | <0.0001^#^ | 13.8 (9.6-18.0) | 22.9 (16.1-29.7) | 0.0011 | | |
| West BANK | 5.7 (4.0-7.4) | 8.3 (5.2-11.4) | 3.3 (2.1-4.4) | 0.0002 | 5.6 (3.3-8.0) | 5.8 (2.3-9.3) | 0.93 | | |
| Yemen | 10.6 (7.7-13.5) | 14.8 (11.7-17.8) | 4.3 (2.3-6.3) | <0.0001^#^ | 11.1 (7.1-15.1) | 10.2 (7.3-13.1) | 0.65 | | |
| **Europe** |  |  |  |  |  |  |  | | |
| Albania | 3.8 (3.3-4.3) | 5.5 (4.5-6.6) | 1.9 (1.3-2.5) | <0.0001^#^ | 2.8 (2.1-3.4) | 5.1 (4.1-6.0) | 0.0003 | | |
| Azerbaijan | 1.9 (1.2-2.7) | 2.7 (1.5-3.8) | 1.1 (0.6-1.6) | 0.0024 | 1.9 (1.1-2.6) | 2.0 (0.5-3.5) | 0.88 | | |
| Belarus | 0.8 (0.4-1.2) | 1.1 (0.5-1.8) | 0.4 (0.0-0.8) | 0.045 | 0.6 (0.1-1.0) | 1.1 (0.3-2.0) | 0.20 | | |
| Bosnia and Herzegovina | 2.3 (1.8-2.7) | 2.9 (2.2-3.6) | 1.5 (1.0-2.1) | 0.0032 | 1.7 (1.3-2.0) | 3.3 (2.2-4.3) | 0.0004 | | |
| Bulgaria | 2.7 (1.4-3.9) | 3.6 (1.6-5.6) | 1.6 (0.9-2.4) | 0.0031 | 2.5 (1.4-3.6) | 2.8 (1.2-4.4) | 0.64 | | |
| Croatia (Hrvatska) | 2.0 (1.2-2.7) | 2.8 (1.7-3.9) | 1.2 (0.5-1.9) | 0.0024 | 1.8 (0.8-2.8) | 2.3 (1.3-3.2) | 0.51 | | |
| Czech Republic | 4.7 (3.6-5.9) | 6.6 (4.8-8.4) | 2.7 (1.8-3.7) | <0.0001^#^ | 3.4 (2.3-4.6) | 7.7 (5.6-9.8) | <0.0001^#^ | | |
| Finland | 7.1 (5.9-8.3) | 12.4 (10.3-14.5) | 1.6 (1.0-2.2) | <0.0001^#^ | 5.3 (4.5-6.2) | 10.5 (7.8-13.1) | | | <0.0001^#^ |
| Georgia | 3.4 (1.5-5.4) | 4.0 (1.8-6.2) | 2.9 (0.6-5.1) | 0.34 | 3.7 (1.6-5.9) | 3.0 (0.9-5.1) | | | 0.43 |
| Greece | 1.5 (1.2-1.9) | 2.0 (1.5-2.5) | 1.0 (0.5-1.5) | 0.013 | 1.3 (1.0-1.6) | 2.1 (1.2-3.0) | | | 0.032 |
| Italy | 1.7 (1.0-2.3) | 1.9 (0.7-3.1) | 1.4 (0.4-2.4) | 0.60 | 1.0 (0.3-1.8) | 2.9 (1.7-4.1) | | | 0.0079 |
| Kazakhstan | 0.5 (0.2-0.8) | 0.7 (0.2-1.2) | 0.4 (0.2-0.6) | 0.36 | 0.6 (0.2-0.9) | 0.4 (0.0-1.0) | | | 0.65 |
| Kosovo | 2.7 (2.2-3.3) | 3.8 (3.0-4.7) | 1.5 (1.0-2.0) | <0.0001^#^ | 2.6 (2.0-3.2) | 3.5 (1.9-5.1) | | | 0.26 |
| Kyrgyzstan | 2.5 (1.5-3.4) | 4.3 (2.6-6.1) | 0.6 (0.2-0.9) | <0.0001^#^ | 2.0 (1.2-2.9) | 3.2 (1.6-4.8) | | | 0.11 |
| Latvia | 5.4 (3.8-6.9) | 7.0 (4.8-9.1) | 3.7 (2.4-5.0) | 0.0002 | 3.4 (2.4-4.4) | 8.8 (5.6-12.0) | | | <0.0001^#^ |
| Lithuania | 3.0 (1.9-4.2) | 4.3 (2.3-6.2) | 1.8 (1.1-2.5) | 0.0005 | 2.3 (1.3-3.2) | 3.9 (2.0-5.8) | | | 0.037 |
| Macedonia | 2.1 (1.5-2.6) | 2.4 (1.7-3.0) | 1.7 (1.1-2.3) | 0.070 | 2.0 (1.4-2.6) | 2.1 (1.1-3.1) | | | 0.94 |
| Malta | 1.1 (0.5-1.6) | 1.7 (0.8-2.7) | 0.2 (0.0-0.5) | 0.0072 | 1.0 (0.3-1.7) | 1.2 (0.2-2.1) | | | 0.71 |
| Montenegro | 2.2 (1.7-2.7) | 2.6 (1.9-3.4) | 1.7 (1.1-2.3) | 0.047 | 1.9 (1.4-2.5) | 2.7 (2.0-3.4) | | | 0.096 |
| Poland | 6.4 (4.9-7.9) | 9.0 (6.8-11.3) | 3.7 (2.3-5.1) | <0.0001^#^ | 4.7 (3.2-6.1) | 7.5 (5.8-9.3) | | | 0.0003 |
| Portugal | 3.9 (3.2-4.6) | 4.5 (3.5-5.4) | 3.3 (2.7-4.0) | 0.011 | 3.9 (3.2-4.7) | 3.7 (2.5-5.0) | | | 0.78 |
| Republic of Moldova | 1.8 (1.3-2.3) | 2.3 (1.5-3.1) | 1.3 (0.6-2.0) | 0.062 | 1.5 (0.8-2.2) | 2.2 (1.3-3.1) | | | 0.28 |
| Romania | 6.9 (5.8-8.1) | 7.9 (6.4-9.4) | 5.9 (4.9-7.0) | 0.0032 | 6.8 (5.6-8.0) | 7.5 (5.5-9.5) | | | 0.51 |
| Russian Federation | 2.9 (1.8-4.0) | 4.0 (2.5-5.5) | 1.8 (0.7-2.9) | 0.0064 | 2.2 (1.2-3.3) | 4.3 (3.1-6.4) | | | 0.054 |
| San Marino | 0.7 (0.0-1.3) | 0.9 (0.0-1.9) | 0.3 (0.0-1.0) | 0.36 | 0.6 (0.0-1.3) | 0.8 (0.0-1.9) | | | 0.70 |
| Serbia | 1.8 (1.4-2.2) | 2.3 (1.6-2.9) | 1.4 (0.8-1.9) | 0.041 | 1.6 (1.1-2.2) | 2.0 (1.4-2.7) | | | 0.33 |
| Slovakia | 3.8 (2.9-4.7) | 5.2 (3.7-6.8) | 2.3 (1.5-3.1) | <0.0001^#^ | 3.0 (2.2-3.8) | 5.4 (3.6-7.1) | | | 0.0003 |
| Slovenia | 3.5 (2.4-4.6) | 5.2 (3.0-7.4) | 1.6 (0.8-2.4) | 0.0010 | 2.8 (1.5-4.1) | 4.2 (2.2-6.3) | | | 0.25 |
| Tajikistan | 1.3 (0.9-1.6) | 1.7 (1.0-2.3) | 0.8 (0.4-1.3) | 0.054 | 1.3 (0.7-2.0) | 1.2 (0.6-1.7) | | | 0.75 |
| Turkmenistan | 0.2 (0.1-0.3) | 0.2 (0.0-0.3) | 0.2 (0.0-0.4) | 0.81 | 0.2 (0.0-0.5) | 0.2 (0.0-0.3) | | | 0.62 |
| Ukraine | 2.8 (1.9-3.8) | 2.8 (1.9-3.7) | 2.9 (1.6-4.2) | 0.85 | 2.6 (1.6-3.6) | 5.9 (3.0-8.8) | | | 0.013 |
| **South-East Asia** |  |  |  |  |  |  | | |  |
| Bangladesh | 4.4 (2.5-6.4) | 5.1 (2.3-7.9) | 3.2 (0.9-5.4) | 0.34 | 3.8 (2.3-5.4) | 9.2 (6.5-11.9) | | | 0.0012 |
| Bhutan | 21.7 (18.5-25.0) | 26.3 (23.0-29.6) | 17.9 (13.9-21.9) | <0.0001^#^ | 15.4 (11.5-19.2) | 30.0 (25.2-34.7) | | | <0.0001^#^ |
| Indonesia | 2.1 (1.5-2.7) | 3.0 (2.1-3.8) | 1.3 (0.8-1.8) | <0.0001^#^ | 1.8 (1.3-2.2) | 4.5 (2.0-6.9) | | | 0.0010 |
| Maldives | 6.3 (4.8-7.9) | 3.3 (2.2-4.3) | 9.9 (7.3-12.4) | <0.0001^#^ | 6.7 (4.6-8.8) | 5.9 (3.7-8.2) | | | 0.63 |
| Myanmar | 7.5 (5.7-9.3) | 13.2 (10.2-16.2) | 2.7 (1.4-3.9) | <0.0001^#^ | 5.8 (4.3-7.4) | 9.8 (7.3-12.2) | | | 0.0003 |
| Nepal | 4.4 (3.1-5.6) | 4.3 (2.9-5.8) | 4.4 (2.8-6.0) | 0.95 | 4.3 (2.6-6.0) | 4.4 (3.0-5.8) | | | 0.94 |
| Sri Lanka | 29.7 (20.6-38.9) | 34.7 (24.3-45.2) | 24.7 (11.7-37.7) | 0.21 | 27.8 (19.4-36.2) | 33.7 (19.0-48.4) | | | 0.34 |
| Thailand | 3.1 (2.1-4.2) | 5.7 (3.8-7.6) | 0.5 (0.1-0.9) | <0.0001^#^ | 2.2 (1.2-3.2) | 6.0 (3.0-9.0) | | | 0.0013 |
| Timor-Leste | 8.7 (6.6-10.8) | 7.8 (4.4-11.1) | 9.7 (7.0-12.4) | 0.38 | 6.8 (3.9-9.6) | 11.6 (9.4-13.8) | | | 0.018 |
| **Western Pacific** |  |  |  |  |  |  | | |  |
| Brunei Darussalam | 0.9 (0.3-1.4) | 1.1 (0.5-1.6) | 0.7 (0.0-1.5) | 0.39 | 1.2 (0.5-1.8) | 0.5 (0.0-1.2) | | | 0.20 |
| Cambodia | 1.5 (1.0-2.1) | 1.7 (0.9-2.6) | 1.3 (0.6-2.1) | 0.54 | 1.0 (0.4-1.5) | 2.0 (1.2-2.7) | | | 0.029 |
| China | 1.0 | 1.3 | 0.6 | — | — | — | | | — |
| Cook Islands | 4.2 (2.4-6.0) | 5.8 (2.7-8.8) | 2.8 (0.8-4.9) | 0.11 | 3.0 (0.6-5.3) | 5.2 (2.5-7.8) | | | 0.24 |
| Fiji | 2.2 (1.4-3.0) | 2.9 (1.8-4.1) | 1.4 (0.6-2.2) | 0.024 | 2.1 (1.0-3.2) | 2.2 (1.2-3.2) | | | 0.87 |
| Guam (United States) ^a^ | 16.6 (14.4-18.8) | 17.0 (14.2-19.7) | 16.2 (13.0-19.3) | 0.69 | 13.7 (11.1-16.3) | 19.9 (16.5-23.3) | | | 0.0020 |
| Kiribati | 51.6 (46.3-57.0) | 53.9 (47.9-60.0) | 49.5 (43.6-55.4) | 0.11 | 43.7 (38.4-49.0) | 62.9 (58.5-67.2) | | | <0.0001^#^ |
| Laos | 1.9 (1.3-2.5) | 2.4 (1.6-3.2) | 1.4 (0.9-1.9) | 0.0084 | 1.7 (1.1-2.3) | 2.1 (1.4-2.8) | | | 0.26 |
| Macao (China) ^a^ | 1.5 (1.0-2.0) | 1.6 (0.9-2.2) | 1.4 (0.7-2.1) | 0.68 | 1.7 (0.7-2.7) | 1.3 (0.4-2.3) | | | 0.70 |
| Marshall Islands | 25.1 (22.4-27.7) | 34.0 (30.3-37.7) | 18.0 (15.0-20.9) | <0.0001^#^ | 21.7 (17.7-25.8) | 27.3 (24.2-30.4) | | | 0.025 |
| Micronesia | 34.6 (33.0-36.2) | 41.5 (39.1-43.9) | 28.7 (26.7-30.8) | <0.0001^#^ | 30.6 (28.8-32.4) | 44.3 (41.3-47.4) | | | <0.0001^#^ |
| Mongolia | 7.4 (6.5-8.2) | 10.5 (9.1-11.8) | 4.5 (3.6-5.3) | <0.0001^#^ | 7.0 (6.0-8.0) | 8.3 (6.8-9.7) | | | 0.16 |
| Northern Mariana Islands | 22.0 (19.0-24.9) | 27.2 (24.1-30.4) | 16.5 (11.2-21.8) | 0.0028 | 19.1 (15.9-22.3) | 30.9 (26.4-35.5) | | | <0.0001^#^ |
| Palau | 19.9 (17.1-22.7) | 20.9 (17.4-24.3) | 19.0 (15.6-22.5) | 0.37 | 16.7 (14.0-19.4) | 25.2 (19.8-30.5) | | | 0.0009 |
| Papua New Guinea | 12.7 (9.8-15.6) | 13.6 (9.9-17.3) | 11.8 (8.6-15.0) | 0.35 | 10.6 (7.1-14.0) | 15.5 (12.0-18.9) | | | 0.027 |
| Philippines | 2.4 (1.6-3.2) | 2.8 (1.8-3.7) | 2.1 (1.2-2.9) | 0.099 | 2.3 (1.3-3.4) | 2.5 (1.3-3.7) | | | 0.83 |
| Samoa | 2.0 (1.1-3.0) | 2.7 (1.0-4.4) | 1.4 (0.7-2.1) | 0.083 | 1.9 (0.7-3.1) | 2.1 (1.0-3.3) | | | 0.76 |
| South Korea | 0.9 (0.4-1.5) | 1.0 (0.4-1.6) | 0.8 (0.2-1.4) | 0.53 | 0.9 (0.4-1.4) | 1.0 (0.0-1.9) | | | 0.83 |
| Tokelau | 0.0 | 0.0 | 0.0 | — | 0.0 | 0.0 | | | — |
| Tonga | 17.4 (10.6-24.2) | 18.9 (13.2-24.6) | 16.3 (7.7-24.9) | 0.46 | 15.0 (10.2-19.9) | 21.0 (8.7-33.4) | | | 0.21 |
| Tuvalu | 3.2 (1.8-4.6) | 3.5 (1.0-6.0) | 3.0 (1.5-4.5) | 0.72 | 3.9 (1.9-5.8) | 2.2 (0.5-3.9) | | | 0.21 |
| Vanuatu | 5.1 (3.5-6.7) | 5.3 (2.9-7.7) | 4.9 (3.0-6.8) | 0.76 | 4.3 (2.1-6.5) | 5.6 (3.6-7.6) | | | 0.40 |
| Viet Nam | 1.0 (0.6-1.5) | 1.6 (0.8-2.5) | 0.5 (0.1-0.8) | 0.0040 | 0.8 (0.2-1.3) | 1.5 (0.8-2.2) | | | 0.090 |
| *P*-value (across countries) | <0.0001^#^ | <0.0001^#^ | <0.0001^#^ |  | <0.0001^#^ | <0.0001^#^ | | |  |

Prevalence estimates are weighted at country level.

CI: confidence intervals.

UNRWA: United Nations Relief and Works Agency.

^a^ Macao is the special administrative region of China; Guam is an overseas territory of the United States; Gaza and West bank are territories of Palestine.

^#^ *P* value <0.00018 (0.05/276, which is the Bonferroni’s corrected critical significance level).

**Table S4. Trends in the prevalence of current smokeless tobacco use among adolescents aged 12-16 years in 100 countries from 1999 to 2019 by country/territory**

| Country/territory | Representativeness | Survey year | First year,  %(95%CI) | Last year,  %(95%CI) | Total absolute change, % | Absolute change/5-years, % | *P* for trend |
| --- | --- | --- | --- | --- | --- | --- | --- |
| **Africa** |  |  |  |  |  |  |  |
| Cameroon | Subnational | 2008, 2014 | 5.0 (3.4-6.7) | 3.6 (2.3-5.0) | -1.4 | -1.2 | <0.0001^#^ |
| Congo | National | 2006, 2009, 2019 | 18.0 (12.8-23.2) | 6.9 (4.9-8.9) | -11.1 | -4.3 | <0.0001^#^ |
| Gambia | Subnational | 2008, 2017 | 21.4 (17.3-25.5) | 1.7 (1.3-2.0) | -19.7 | -10.9 | <0.0001^#^ |
| Ghana | National | 2006, 2009, 2017 | 19.1 (14.6-23.5) | 3.4 (2.3-4.5) | -15.7 | -7.1 | <0.0001^#^ |
| Madagascar | National | 2008, 2018 | 5.1 (0.9-9.4) | 4.2 (2.6-5.8) | -0.9 | -0.5 | <0.0001^#^ |
| Malawi | National | 2005, 2009 | 3.8 (2.1-5.5) | 8.5 (5.2-11.9) | 4.7 | 5.9 | <0.0001^#^ |
| Mauritania | National | 2001, 2006, 2009, 2018 | 17.7 (15.2-20.2) | 6.7 (5.6-7.7) | -11.0 | -3.2 | <0.0001^#^ |
| Senegal | National | 2007, 2013 | 8.2 (3.8-12.6) | 8.3 (3.5-13.2) | 0.1 | 0.1 | 0.10 |
| Seychelles | National | 2007, 2015 | 5.4 (3.7-7.1) | 1.8 (1.1-2.4) | -3.6 | -2.3 | <0.0001^#^ |
| Sierra Leone | Subnational | 2008, 2017 | 18.4 (15.2-21.7) | 6.3 (2.9-9.6) | -12.1 | -6.7 | <0.0001^#^ |
| South Africa | National | 1999, 2002, 2008, 2011 | 12.6 (8.2-17.1) | 8.2 (6.4-10.0) | -4.4 | -1.8 | <0.0001^#^ |
| Swaziland | National | 2001, 2009 | 0.8 (0.5-1.2) | 5.9 (3.9-7.9) | 5.1 | 3.2 | <0.0001^#^ |
| Togo | National | 2007, 2013, 2019 | 6.5 (5.3-7.7) | 2.0 (1.2-2.9) | -4.5 | -1.9 | <0.0001^#^ |
| Uganda | National | 2007, 2011, 2018 | 10.2 (8.6-11.8) | 6.2 (4.8-7.7) | -4.0 | -1.8 | <0.0001^#^ |
| United Republic of Tanzania | Subnational | 2008, 2016 | 6.2 (4.7-7.8) | 5.1 (3.5-6.6) | -1.1 | -0.7 | <0.0001^#^ |
| Zambia | Subnational | 2007, 2011 | 16.6 (13.3-19.9) | 14.7 (13.3-16.0) | -1.9 | -2.4 | 0.0089 |
| Zimbabwe | Subnational | 2008, 2014 | 5.3 (3.3-7.3) | 31.4 (25.8-37.0) | 26.1 | 21.8 | <0.0001^#^ |
| **Americas** |  |  |  |  |  |  |  |
| Antigua and Barbuda | National | 2009, 2017 | 7.0 (5.5-8.4) | 2.0 (1.3-2.7) | -5.0 | -3.1 | <0.0001^#^ |
| Argentina | National | 2007, 2012, 2018 | 4.3 (3.5-5.2) | 1.5 (0.7-2.3) | -2.8 | -1.3 | <0.0001^#^ |
| Bahamas | National | 2009, 2013 | 6.6 (5.3-7.9) | 2.3 (1.0-3.6) | -4.3 | -5.4 | <0.0001^#^ |
| Barbados | National | 2007, 2013 | 10.0 (8.3-11.6) | 3.1 (2.1-4.0) | -6.9 | -5.8 | <0.0001^#^ |
| Belize | National | 2008, 2014 | 6.1 (4.8-7.5) | 2.8 (2.0-3.7) | -3.3 | -2.8 | <0.0001^#^ |
| Bolivia | National | 2012, 2018 | 5.1 (2.7-7.6) | 3.1 (2.2-3.9) | -2.0 | -1.7 | <0.0001^#^ |
| Brazil | Subnational | 2009, 2011, 2012 | 7.4 (6.0-8.8) | 3.9 (2.4-5.3) | -3.5 | -5.8 | <0.0001^#^ |
| Dominican Republic | National | 2011, 2016 | 11.1 (8.0-14.2) | 4.5 (2.6-6.3) | -6.6 | -6.6 | <0.0001^#^ |
| El Salvador | National | 2009, 2015 | 3.7 (2.9-4.5) | 2.0 (1.3-2.7) | -1.7 | -1.4 | <0.0001^#^ |
| Grenada | National | 2009, 2016 | 8.1 (6.8-9.4) | 1.6 (1.1-2.2) | -6.5 | -4.6 | <0.0001^#^ |
| Guyana | National | 2010, 2015 | 7.6 (5.5-9.8) | 6.6 (4.1-9.1) | -1.0 | -1.0 | <0.0001^#^ |
| Jamaica | National | 2010, 2017 | 9.0 (6.8-11.2) | 2.5 (1.7-3.4) | -6.5 | -4.6 | <0.0001^#^ |
| Mexico | Subnational | 2008, 2011 | 4.6 (4.2-5.0) | 5.0 (4.0-6.1) | 0.4 | 0.7 | <0.0001^#^ |
| Nicaragua | National | 2014, 2019 | 4.0 (3.3-4.8) | 3.3 (2.8-3.9) | -0.7 | -0.7 | <0.0001^#^ |
| Panama | National | 2008, 2012, 2017 | 3.7 (2.7-4.7) | 2.5 (1.8-3.2) | -1.2 | -0.7 | <0.0001^#^ |
| Paraguay | National | 2014, 2019 | 1.6 (0.7-2.4) | 1.7 (1.1-2.3) | 0.09 | 0.1 | 0.0041 |
| Peru | National | 2007, 2014, 2019 | 4.6 (3.4-5.7) | 1.7 (1.3-2.1) | -2.9 | -1.2 | <0.0001^#^ |
| Saint Lucia | National | 2011, 2017 | 6.4 (4.8-8.0) | 7.1 (5.2-9.1) | 0.7 | 0.6 | 0.069 |
| Saint Vincent and the Grenadines | National | 2011, 2018 | 6.8 (5.2-8.3) | 5.8 (4.2-7.4) | -1.0 | -0.7 | 0.042 |
| Suriname | National | 2009, 2016 | 5.0 (3.7-6.3) | 2.9 (2.1-3.7) | -2.1 | -1.5 | <0.0001^#^ |
| Trinidad and Tobago | National | 2007, 2011, 2017 | 6.7 (5.5-7.9) | 7.2 (6.2-8.3) | 0.5 | 0.3 | <0.0001^#^ |
| United States of America | National | 1999, 2000, 2002, 2004, 2006, 2009, 2011, 2012, 2013, 2014, 2015, 2016, 2017, 2018, 2019 | 4.9 (3.7-6.1) | 2.5 (1.8-3.1) | -2.4 | -0.6 | <0.0001^#^ |
| Venezuela | National | 1999, 2010 | 5.1 (4.0-6.2) | 5.2 (4.3-6.1) | 0.1 | 0.05 | <0.0001^#^ |
| **Eastern Mediterranean** |  |  |  |  |  |  |  |
| Afghanistan | Subnational | 2010, 2017 | 8.2 (0.3-16.2) | 4.4 (3.0-5.8) | -3.8 | -2.7 | <0.0001^#^ |
| Djibouti | National | 2009, 2013 | 12.8 (9.7-16.0) | 15.6 (12.8-18.4) | 2.8 | 3.5 | <0.0001^#^ |
| Gaza Strip | Subnational | 2013, 2019 | 5.2 (3.2-7.3) | 4.2 (1.6-6.8) | -1.0 | -0.8 | <0.0001^#^ |
| Iraq | National | 2014, 2019 | 4.0 (2.3-5.7) | 5.9 (3.8-8.0) | 1.9 | 1.9 | <0.0001^#^ |
| Jordan | National | 2009, 2014 | 4.3 (2.9-5.7) | 9.0 (5.6-12.3) | 4.7 | 4.7 | <0.0001^#^ |
| Morocco | National | 2010, 2016 | 7.5 (4.8-10.2) | 3.8 (2.2-5.4) | -3.7 | -3.1 | <0.0001^#^ |
| Oman | National | 2002, 2010, 2016 | 6.7 (4.2-9.1) | 6.9 (3.9-9.9) | 0.2 | 0.1 | 0.53 |
| Pakistan | Subnational | 2008, 2013 | 10.4 (7.1-13.7) | 3.7 (2.8-4.6) | -6.7 | -6.7 | <0.0001^#^ |
| Qatar | National | 2007, 2013, 2018 | 7.3 (5.5-9.1) | 13.0 (9.5-16.5) | 5.7 | 2.6 | <0.0001^#^ |
| Sudan | National | 2005, 2009 | 3.0 (1.6-4.5) | 3.8 (2.1-5.5) | 0.8 | 1.0 | <0.0001^#^ |
| Tunisia | National | 2007, 2010, 2017 | 4.7 (3.5-5.8) | 2.5 (1.7-3.2) | -2.2 | -1.1 | <0.0001^#^ |
| UNRWA GAZA (Palestine) ^a^ | Regional | 2008, 2013 | 8.5 (7.0-9.9) | 13.1 (9.0-17.2) | 4.6 | 4.6 | <0.0001^#^ |
| UNRWA Jordan | Regional | 2008, 2014 | 7.8 (5.6-9.9) | 13.7 (9.1-18.3) | 5.9 | 4.9 | <0.0001^#^ |
| UNRWA Lebanon | Regional | 2008, 2013 | 6.3 (4.6-8.0) | 12.5 (9.2-15.9) | 6.2 | 6.2 | <0.0001^#^ |
| UNRWA west bank (Palestine) ^a^ | Regional | 2008, 2014 | 9.0 (7.3-10.6) | 15.7 (11.2-20.2) | 6.7 | 5.6 | <0.0001^#^ |
| Yemen | National | 2008, 2014 | 10.5 (6.9-14.0) | 10.6 (7.6-13.6) | 0.1 | 0.1 | 0.013 |
| **Europe** |  |  |  |  |  |  |  |
| Albania | National | 2004, 2009, 2015 | 5.0 (3.9-6.1) | 3.8 (3.3-4.3) | -1.2 | -0.5 | <0.0001^#^ |
| Azerbaijan | National | 2011, 2016 | 2.3 (1.4-3.2) | 1.9 (1.2-2.7) | -0.4 | -0.4 | <0.0001^#^ |
| Belarus | National | 2004, 2015 | 3.2 (2.6-3.8) | 0.8 (0.4-1.2) | -2.4 | -1.1 | <0.0001^#^ |
| Bosnia and Herzegovina | National | 2008, 2013 | 1.6 (1.3-1.9) | 2.4 (1.8-2.9) | 0.8 | 0.8 | <0.0001^#^ |
| Croatia (Hrvatska) | National | 2003, 2007, 2011, 2016 | 1.5 (1.0-2.0) | 2.0 (1.2-2.8) | 0.5 | 0.2 | <0.0001^#^ |
| Estonia | National | 2003, 2007 | 2.2 (1.5-2.8) | 7.3 (5.0-9.5) | 5.1 | 6.4 | <0.0001^#^ |
| Georgia | National | 2003, 2014, 2017 | 1.8 (1.3-2.2) | 3.4 (1.4-5.4) | 1.6 | 0.6 | <0.0001^#^ |
| Greece | National | 2005, 2013 | 8.1 (6.6-9.6) | 1.5(1.2-1.9) | -6.6 | -4.1 | <0.0001^#^ |
| Kazakhstan | National | 2004, 2009, 2014 | 2.5 (2.0-3.1) | 0.5 (0.2-0.8) | -2.0 | -1.0 | <0.0001^#^ |
| Kosovo | National | 2004, 2016 | 5.0 (3.9-6.2) | 2.7 (2.2-3.3) | -2.3 | -1.0 | <0.0001^#^ |
| Kyrgyzstan | National | 2004, 2008, 2014, 2019 | 1.8 (0.7-2.8) | 2.5 (1.5-3.4) | 0.7 | 0.2 | <0.0001^#^ |
| Latvia | National | 2007, 2011, 2014, 2019 | 10.9 (7.4-14.4) | 5.4 (3.8-6.9) | -5.5 | -2.3 | <0.0001^#^ |
| Lithuania | National | 2014, 2018 | 2.5 (1.7-3.2) | 3.0 (1.8-4.2) | 0.5 | 0.6 | <0.0001^#^ |
| Macedonia | National | 2003, 2008, 2016 | 2.0 (1.2-2.8) | 2.1 (1.5-2.6) | 0.1 | 0.04 | 0.10 |
| Montenegro | National | 2004, 2008, 2014, 2018 | 1.2 (0.8-1.7) | 2.2 (1.7-2.6) | 1.0 | 0.4 | <0.0001^#^ |
| Poland | National | 2003, 2016 | 1.8 (1.3-2.4) | 6.4 (4.9-8.0) | 4.6 | 1.8 | <0.0001^#^ |
| Republic of Moldova | National | 2004, 2008, 2013, 2019 | 3.9 (3.1-4.7) | 1.8 (1.4-2.2) | -2.1 | -0.7 | <0.0001^#^ |
| Romania | National | 2004, 2017 | 3.2 (2.2-4.2) | 6.9 (5.8-8.1) | 3.7 | 1.4 | <0.0001^#^ |
| Russian Federation | Subnational | 2004, 2015 | 3.7 (2.9-4.6) | 2.9 (1.8-4.0) | -0.8 | -0.4 | <0.0001^#^ |
| San Marino | National | 2014, 2018 | 0.5 (0.0-1.0) | 0.7 (0.0-1.3) | 0.2 | 0.3 | 0.71 |
| Serbia | National | 2008, 2013, 2017 | 1.6 (1.0-2.2) | 1.8 (1.3-2.4) | 0.2 | 0.1 | <0.0001^#^ |
| Slovenia | National | 2003, 2007, 2011, 2017 | 2.4 (1.7-3.2) | 3.5 (2.3-4.6) | 1.1 | 0.4 | <0.0001^#^ |
| Tajikistan | National | 2004, 2014, 2019 | 3.4 (0.9-6.0) | 1.3 (0.9-1.6) | -2.1 | -0.7 | <0.0001^#^ |
| Ukraine | National | 2005, 2011, 2017 | 1.6 (1.0-2.1) | 2.8 (1.9-3.8) | 1.2 | 0.5 | <0.0001^#^ |
| **South-East Asia** |  |  |  |  |  |  |  |
| Bangladesh | National | 2007, 2013 | 5.2 (2.4-7.9) | 4.4 (2.5-6.4) | -0.8 | -0.7 | <0.0001^#^ |
| Bhutan | National | 2004, 2006, 2009, 2013 | 7.0 (4.8-9.2) | 21.7 (18.3-25.1) | 14.7 | 8.2 | <0.0001^#^ |
| India | National | 2006, 2009 | 9.3 (7.3-11.4) | 8.5 (7.3-9.8) | -0.8 | -1.3 | <0.0001^#^ |
| Indonesia | National | 2009, 2014 | 2.9 (2.2-3.7) | 2.1 (1.5-2.7) | -0.8 | -0.8 | <0.0001^#^ |
| Maldives | National | 2007, 2011 | 1.2 (0.6-1.8) | 6.3 (4.8-7.9) | 5.1 | 6.4 | <0.0001^#^ |
| Myanmar | National | 2004, 2007, 2011, 2016 | 15.9 (13.7-18.1) | 7.5 (5.7-9.3) | -8.4 | -3.5 | <0.0001^#^ |
| Nepal | National | 2001, 2007, 2011 | 8.5 (6.1-10.8) | 4.4 (3.2-5.5) | -4.1 | -2.1 | <0.0001^#^ |
| Sri Lanka | National | 2007, 2011, 2015 | 7.6 (5.5-9.8) | 29.7 (20.5-38.9) | 22.1 | 13.8 | <0.0001^#^ |
| Thailand | National | 2009, 2015 | 5.6 (3.2-7.9) | 3.1 (2.1-4.2) | -2.5 | -2.1 | <0.0001^#^ |
| Timor-Leste | National | 2009, 2013 | 43.0 (37.4-48.6) | 8.7 (6.8-10.6) | -34.3 | -42.9 | <0.0001^#^ |
| **Western Pacific** |  |  |  |  |  |  |  |
| Cambodia | National | 2010, 2016 | 4.2 (3.2-5.3) | 1.5 (0.9-2.1) | -2.7 | -2.3 | <0.0001^#^ |
| Cook Islands | National | 2008, 2016 | 8.1 (5.6-10.6) | 4.2 (2.4-6.0) | -3.9 | -2.4 | 0.0070 |
| Guam (United States) ^a^ | National | 2011, 2014, 2017 | 13.0 (10.5-15.5) | 16.6 (14.4-18.8) | 3.6 | 3.0 | <0.0001^#^ |
| Kiribati | National | 2009, 2018 | 32.5 (29.0-36.1) | 51.6 (46.2-57.1) | 19.1 | 10.6 | <0.0001^#^ |
| Macao (China) ^a^ | Regional | 2010, 2015 | 2.3 (1.7-2.9) | 1.5 (1.0-2.0) | -0.8 | -0.8 | <0.0001^#^ |
| Mongolia | National | 2007, 2014 | 13.6 (4.6-22.5) | 7.4 (6.5-8.3) | -6.2 | -4.4 | <0.0001^#^ |
| Palau | National | 2013, 2017 | 19.8 (17.4-22.3) | 19.9 (17.4-22.5) | 0.1 | 0.1 | 0.96 |
| Papua New Guinea | National | 2007, 2016 | 18.8 (16.6-21.0) | 12.7 (9.0-16.3) | -6.1 | -3.4 | <0.0001^#^ |
| Philippines | National | 2007, 2011, 2015 | 5.3 (4.3-6.4) | 2.4 (1.5-3.3) | -2.9 | -1.8 | <0.0001^#^ |
| South Korea | National | 2008, 2013 | 6.1 (5.3-7.0) | 0.9 (0.4-1.5) | -5.2 | -5.2 | <0.0001^#^ |

Country-specific weighted prevalence estimates are presented as % (95% confidence intervals).

UNRWA: United Nations Relief and Works Agency.

^a^ Macao is the special administrative region of China; Guam is an overseas territory of the United States; Gaza and West bank are territories of Palestine.

^#^ *P* value <0.0005(0.05/100, which is the Bonferroni’s corrected critical significance level).

**Table S5. Linear trends per 5 calendar years in the prevalence of current smokeless tobacco use among adolescents aged 12-16 years in 100 countries from 1999 to 2019**

| Group | No. of  countries | Total | Boys | Girls | 12-14 years | 15-16 years |
| --- | --- | --- | --- | --- | --- | --- |
| **Total** | 100 | -0.71 (-1.91 to 0.49) | -0.56 (-1.92 to 0.80) | -0.91 (-2.03 to 0.21) | -1.09 (-2.27 to 0.08) | 0.04 (-1.30 to 1.38) |
| **WHO region** |  |  |  |  |  |  |
| Africa | 17 | -0.82 (-4.39 to 2.76) | -0.44 (-3.93 to 3.05) | -1.20 (-4.86 to 2.46) | -1.37 (-4.63 to 1.90) | -0.10 (-4.42 to 4.22) |
| Americas | 23 | -2.08 (-3.07 to -1.09) | -2.30 (-3.60 to -1.01) | -2.02 (-2.85 to -1.18) | -2.45 (-3.47 to -1.43) | -1.18 (-2.60 to 0.25) |
| Eastern Mediterranean | 16 | 1.30 (-0.64 to 3.23) | 3.08 (-0.04 to 6.21) | -0.40 (-1.71 to 0.92) | 0.88 (-0.82 to 2.59) | 2.46 (-0.69 to 5.61) |
| Europe | 24 | 0.06 (-0.70 to 0.82) | 0.17 (-0.73 to 1.07) | -0.10 (-0.74 to 0.53) | -0.12 (-0.83 to 0.60) | 0.27 (-0.56 to 1.09) |
| South-East Asia | 10 | -2.50 (-13.42 to 8.43) | -3.26 (-15.29 to 8.78) | -1.77 (-11.9 to 8.32) | -2.81 (-13.89 to 8.27) | -1.45 (-12.26 to 9.36) |
| Western Pacific | 10 | -0.66 (-3.95 to 2.63) | -1.66 (-4.57 to 1.26 ) | 0.25 (-3.47 to 3.97) | -1.28 (-4.24 to 1.68) | 0.14 (-4.00 to 4.27) |
| **World Bank income** |  |  |  |  |  |  |
| Low income | 16 | 1.27 (-2.58 to 5.12) | 2.77 (-1.61 to 7.16) | -0.08 (-3.85 to 3.70) | 0.96 (-2.39 to 4.30) | 2.50 (-2.60 to 7.61) |
| Lower-Middle income | 32 | -1.55 (-4.68 to 1.58) | -1.67 (-5.13 to 1.79) | -1.48 (-4.35 to 1.39) | -2.06 (-5.21 to 1.09) | -0.68 (-3.87 to 2.51) |
| Upper-Middle income | 32 | -0.85 (-1.80 to 0.11) | -0.94 (-2.03 to 0.15) | -0.88 (-1.89 to 0.13) | -1.15 (-2.18 to -0.12) | -0.27 (-1.50 to 0.96) |
| High income | 20 | -0.74 (-2.17 to 0.69) | -0.86 (-2.57 to 0.84) | -0.70 (-2.02 to 0.63) | -1.10 (-2.44 to 0.25) | -0.28 (-1.82 to 1.26) |

Data are presented as % (95% confidence intervals).

WHO: World Health Organization.

**
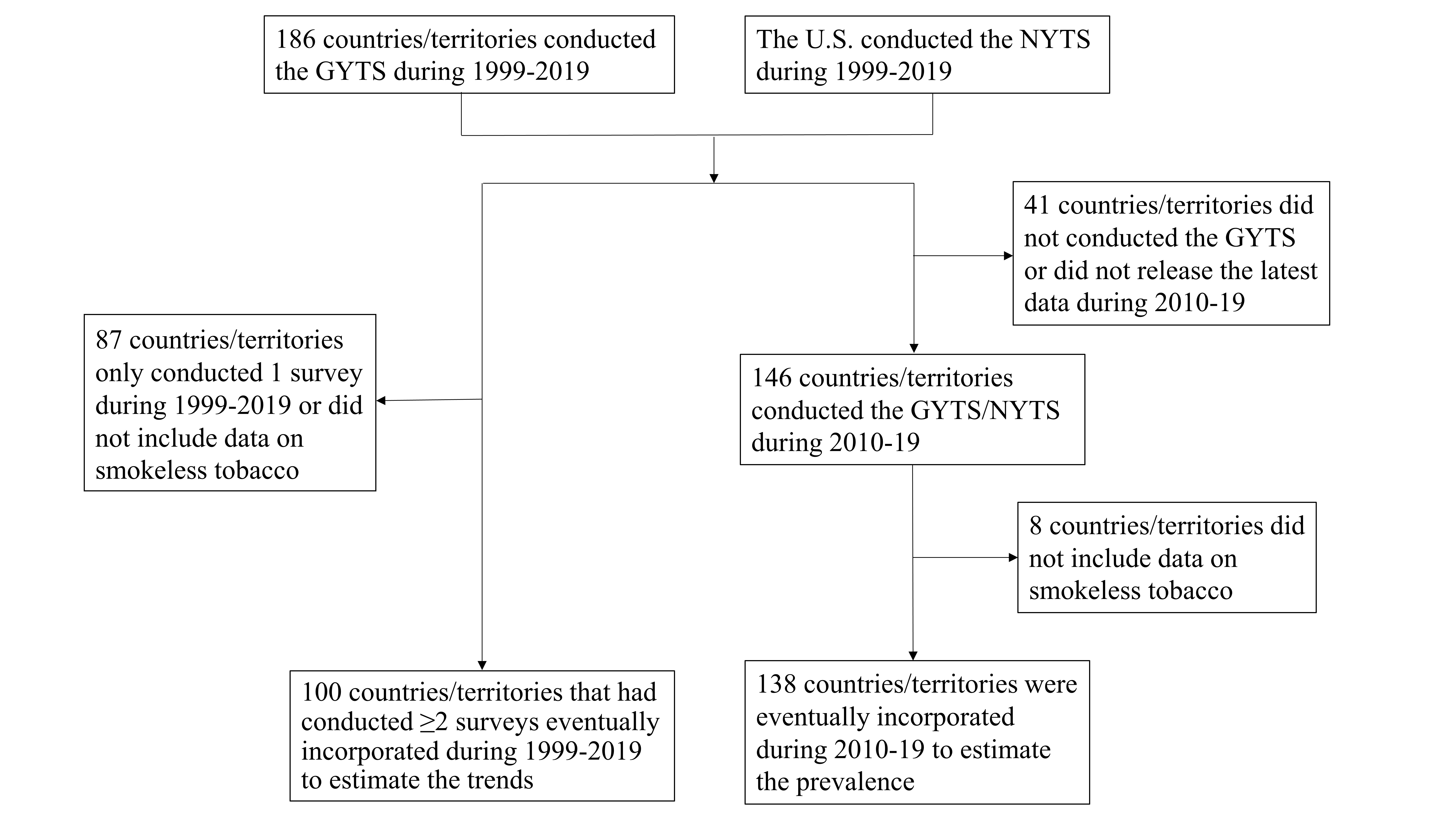
**

**Figure S1:** Flow chart of the inclusion/exclusion of countries/territories


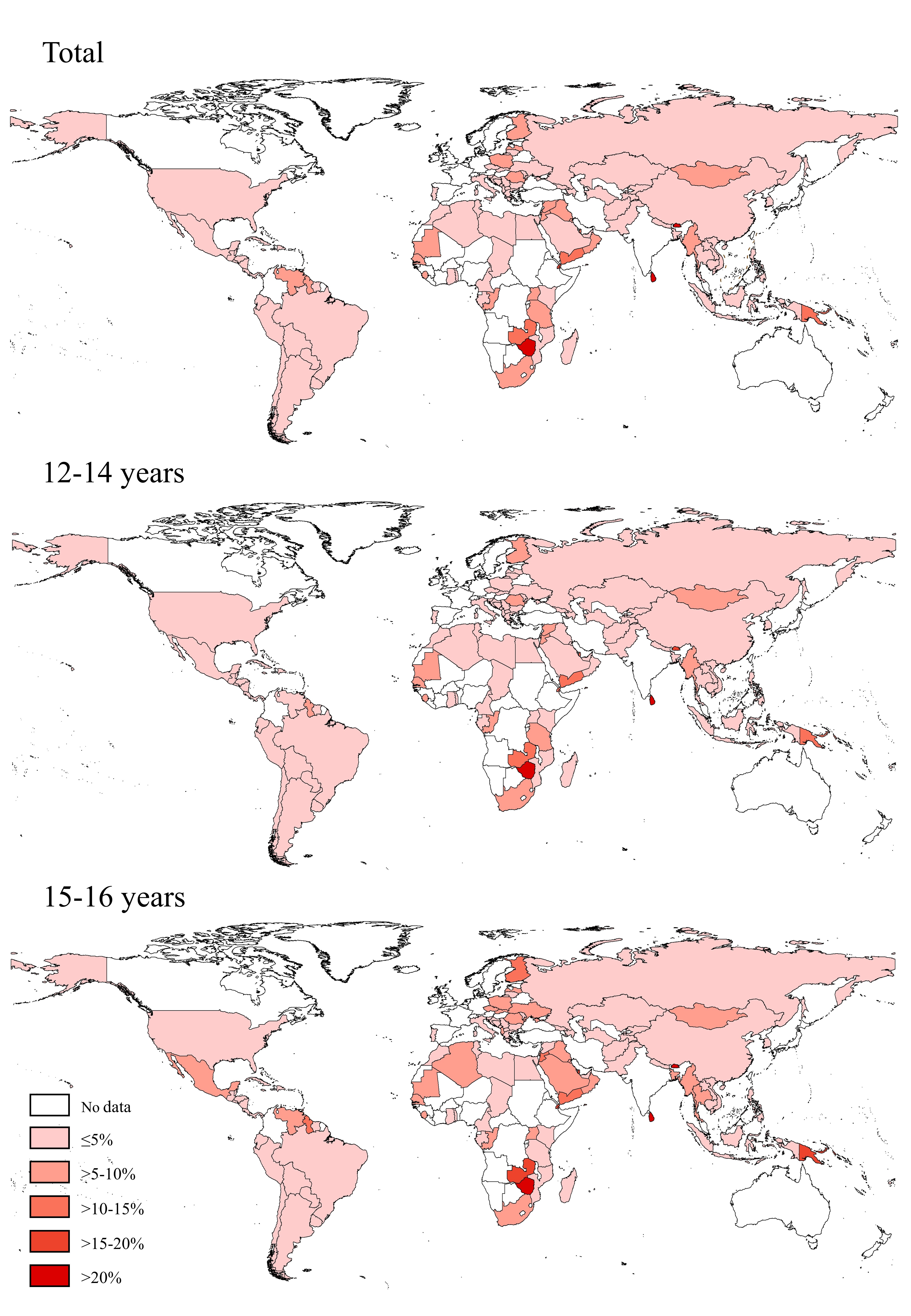


**Figure S2:** Prevalence of current smokeless tobacco use among adolescents aged 12-16 years by age group in 138 countries/territories in 2010-19


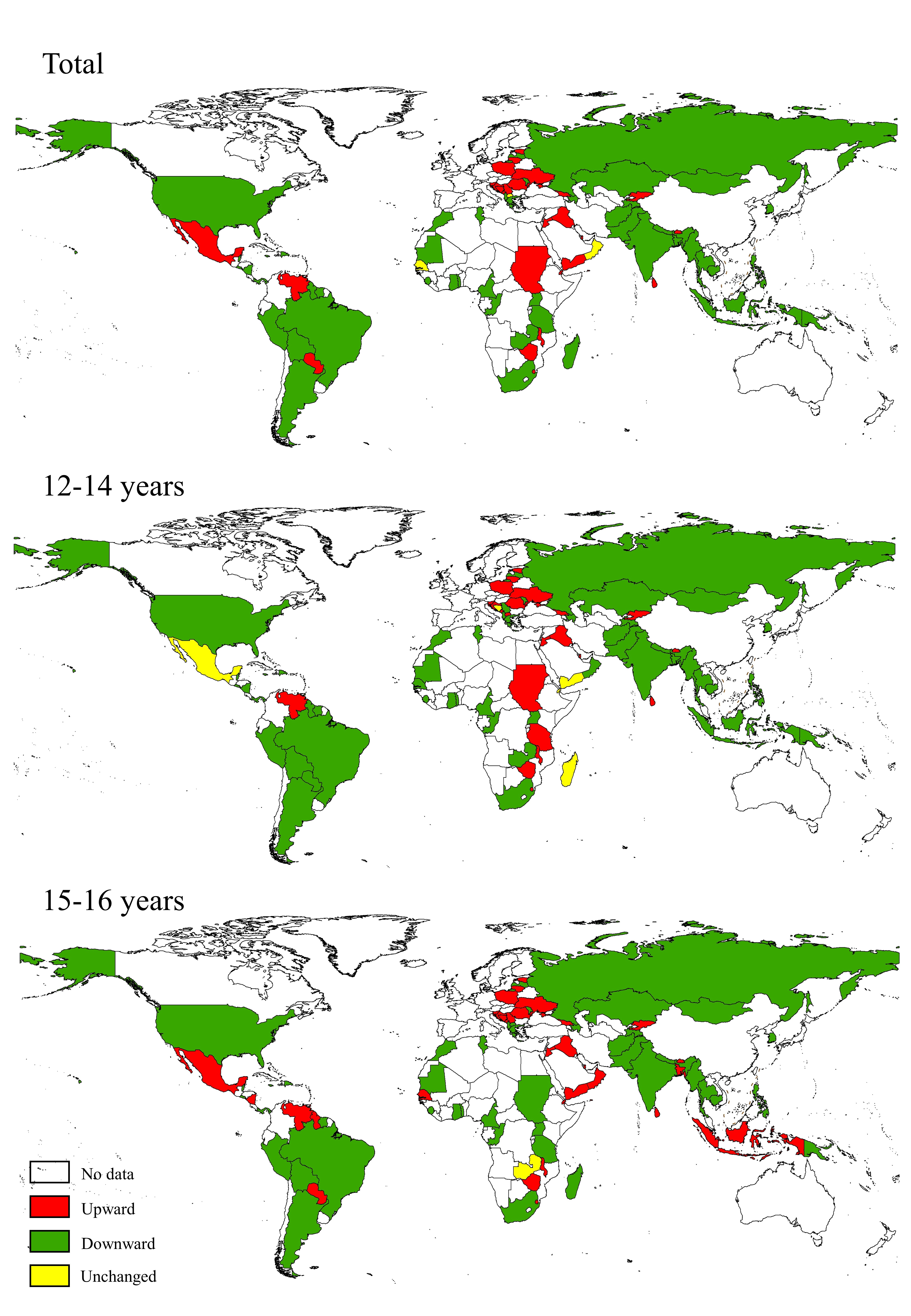


**Figure S3**: Secular trends in current smokeless tobacco use among adolescents aged 12-16 years by age group in 100 countries/territories from 1999 to 2019
